# Decreased neuronal and increased endothelial fractalkine expression are associated with neuroinflammation in Parkinson’s disease and related disorders

**DOI:** 10.3389/fncel.2025.1557645

**Published:** 2025-08-06

**Authors:** Yaping Chu, Ashley S. Harms, Ashley Boehringer, Jeffrey H. Kordower

**Affiliations:** ^1^ASU-Banner Neurodegenerative Disease Research Center, Arizona State University, Tempe, AZ, United States; ^2^Department of Neurology, University of Alabama at Birmingham, Birmingham, AL, United States

**Keywords:** neuron–microglia communication, neuroinflammation, microglia, CD4^+^ T lymphocyte, neurodegenerative disease

## Abstract

**Introduction:**

Chronic neuroinflammation is a persistent feature of individuals with Parkinson’s disease (PD). Dopaminergic neurodegeneration is partially associated with neuroinflammation. Neuron-to-microglia communication via fractalkine (CX3CL1) and its receptor plays a significant role in neuroinflammation. The relationship between fractalkine signaling and neuroinflammatory activities has been studied in animal models of PD, but its role is unclear in human PD. The current study aimed to elucidate the neuron–microglia communication between neuronal and endothelial fractalkine ligand expression, microglial activity, and CD4^+^ T cell infiltration during PD development.

**Methods:**

Brain sections were obtained from age-matched control subjects with no motor deficits (control, *n* = 8), mild motor deficits with nigral Lewy bodies (MMD-LB, *n* = 8) without a clinical diagnosis of PD, individuals with a clinical diagnosis of sporadic PD (*n* = 13), and progressive supranuclear palsy patients (PSP, *n* = 9). We performed quantitative stereological analyses and optical metrology of fractalkine expression in neurons and endothelial cells, and immunoreactivities of microglial and CD4^+^ T cells within the substantia nigra of PD cases. These data were compared with findings seen in age-matched controls as well as MMD-LB and PSP.

**Results:**

In PD, MMD-LB, and PSP postmortem brains, fractalkine expression in remaining nigral neurons was significantly reduced but markedly increased in blood vessel endothelial cells. The density of microglia and CD4^+^ T cells in the substantia nigra was significantly higher in these disorders when compared to controls. The decline in neuronal fractalkine expression was inversely correlated with increases in microglial numbers in the substantia nigra, while higher levels of fractalkine in endothelial cells were positively correlated with CD4^+^ cells infiltrating the injured substantia nigra across groups. Both PD and PSP groups displayed a similar pattern of neuroinflammatory changes.

**Discussion:**

The downregulation of neuronal fractalkine expression caused by protein inclusions, such as abnormal alpha-synuclein or tau, is linked to microglia activation. When activated, microglia release cytokines that further stimulate endothelial cells to express fractalkine. This process recruits peripheral T cells, which infiltrate the injured brain. These findings indicate that the varying levels of neuronal and endothelial fractalkine expression in the substantia nigra contribute to neuroinflammatory activity in both synucleinopathy and tauopathy.

## 1 Introduction

Nigrostriatal dopaminergic neurodegeneration with synucleinopathy results in motor deficits in patients with Parkinson’s disease (PD). Mounting evidence from human brain samples and animal models proves downstream dopaminergic neurodegeneration is partially associated with inflammation in PD (Olanow et al., 2019; Pajares et al., 2020; Harms et al., 2023). Postmortem studies have demonstrated the presence of activated microglia in the substantia nigra (McGeer et al., 1988). Genome-wide association studies (GWAS) have revealed a relationship between polymorphisms of the human leukocyte antigen (HLA) region and late-onset sporadic PD (Hamza et al., 2010; Harms et al., 2021). Positron emission tomography (PET) imaging showed significantly increased mean levels of [^11^C] (*R*)-PK11195 (an *in vivo* marker of activated microglia) binding in the brains of PD patients (Gerhard et al., 2006). The increase of [^11^C] (*R*)-PK11195 binding in the midbrain was correlated with motor deficits, suggesting that microglial activation may play a role in disease pathogenesis (Ouchi et al., 2005). Further, studies in acute and chronic animal models of PD replicate pathological alterations including microgliosis, elevated cytokine expression, lymphocyte invasion, and gradual loss of dopaminergic neurons in the substantia nigra (Ramsey and Tansey, 2014; Allen Reish and Standaert, 2015; Williams et al., 2021; Karikari et al., 2022). Interestingly, phagocytosis of aggregated α-synuclein (α-syn) by microglia induces an immune response to clear pathological agents (Cao et al., 2010). Conversely, immune activation of microglia and cytokine release promote α-syn misfolding and spread (Marogianni et al., 2020; La Vitola et al., 2021; Scheiblich et al., 2021). These data provide clear evidence that immune-mediated neurodegeneration is implicated in the pathogenesis of PD.

Neuron-to-microglia communication plays an important role in development, maintenance, and neuroinflammation (Szepesi et al., 2018; Chamera et al., 2020). Fractalkine (FKN), also known as chemokine C-X3-C motif ligand 1 (CX3CL1), is a transmembrane chemokine expressed primarily by neurons in the central nervous system (Pawelec et al., 2020). Its receptor, CX3CR1, is expressed by tissue-resident macrophages in the central nervous system, including microglia (Wolf et al., 2013). Fractalkine and its receptor participate in neuron-to-microglia communication through CX3CL1/CX3CR1, and this interaction regulates microglial activities (Pabon et al., 2011; Castro-Sánchez et al., 2018; Bartels et al., 2020). *In vivo*, CX3CL1-deficiency aggravates mutant α-syn (A53T) and induces neuroinflammation (Castro-Sánchez et al., 2018). Exogenous CX3CL1 can reduce neurotoxicity and microglia activation in the 6-hydroxydopamine rat model of PD (Pabon et al., 2011).

CX3CL1 is also expressed on vascular endothelial cells, acting as a cell adhesion molecule (Hirono et al., 2020). Some white blood cells, including CD4^+^ helper T cells, are also known to express CX3CR1 (Batista et al., 2020). Endothelial CX3CL1 can bind to CX3CR1 on CD4^+^ helper T cells, facilitating CD4^+^ T cell recruitment to the vessel wall and infiltration into the brain. CX3CL1 expression on endothelial cells can be significantly upregulated by pro-inflammatory cytokines, particularly when elevated levels of inflammation are present (Wu et al., 2017). Whether the infiltration of CD4^+^ T cells into PD brains (Williams et al., 2021) is associated with the upregulating endothelial CX3CL1 is yet unknown.

These studies prompted us to hypothesize that activated microglia in PD brains are associated with the reduction of neuronal CX3CL1 expression, and lymphocyte infiltration into PD brains is related to increases in endothelial CX3CL1 expression. In this regard, the present purpose of this study was to examine CX3CL1 expression in the remaining nigral neurons, as well as endothelial cells, from subjects with sporadic PD, and determine whether alterations in CX3CL1 expression are associated with increased numbers of nigral microglia, and/or CD4^+^ T cell infiltration. To evaluate these hypotheses, the relative levels of CX3CL1 expression were measured in the remaining nigral dopaminergic neurons and endothelial cells, using quantitative immunofluorescence intensity measurements. The number of CX3CL1 immunopositive (CX3CL1-ir) neurons, TMEM119 immunopositive (TMEM119-ir) microglia, and infiltrating CD4^+^ T cells was estimated using unbiased stereology. These data were compared with findings in age-matched controls, individuals with mild motor deficits, as well as individuals with the related disorder, progressive supranuclear palsy (PSP). As PD is primarily a synucleinopathy and PSP a tauopathy, we also compared the alterations in CX3CL1, microglia, and CD4^+^ T cells in both neurodegenerative diseases to determine whether there is a difference between pathologies. In the cohort, we found that CX3CL1 expression was significantly reduced in remaining nigral neurons with α-syn inclusions, or neurofibrillary tangles, and conversely increased in endothelial cells. The number of microglia and CD4^+^ T cells was strongly increased.

## 2 Materials and methods

### 2.1 Subjects

We analyzed brain tissue from age-matched control cases with no motor deficits (control, *n* = 8), clinical and neuropathological diagnoses of mild motor deficits with nigral Lewy bodies (MMD-LB, *n* = 8), sporadic PD (*n* = 13), and PSP (*n* = 9). Each subject signed an informed consent for clinical assessment prior to death and an anatomic gift act for donation of the brain at the time of death. The subjects with MMD-LB and age-matched controls were participants in the Religious Orders Study, a community-based cohort study of chronic conditions of aging, who agreed to brain autopsy at the time of death, and were examined by a neurologist or geriatrician at the Rush Alzheimer’s Disease Center. All adults with sporadic PD and PSP were diagnosed by movement disorder specialists in the Department of Neurological Sciences at Rush University Medical Center.

Subjects with MMD-LB were defined as having mild motor deficits that were insufficient to meet the clinical definition of PD based on UK Brain Bank criteria (Chu et al., 2018, 2024). For PD, inclusion criteria included a history compatible with idiopathic PD, bradykinesia, and one additional cardinal motor sign (resting tremor, rigidity, and gait disturbance), as well as a clinical response to levodopa. For PSP, the criteria rest upon four clinical domains: oculomotor dysfunction, postural instability, akinesia, and cognitive dysfunction. Exclusion criteria included familial PD, the Lewy body variant of Alzheimer’s disease, or the combination of PD and Alzheimer’s disease as defined neuropathologically. The clinical characteristics of these cases are summarized in Table 1. The Human Investigation Committee at Rush University Medical Center approved this study.

**Table 1:** Clinical and postmortem characteristics (mean-standard deviation).

Measure	Control	MMD-LB	PD	PSP
Case number	8	8	13	9
Age at death (years)	85.87 ± 5.93	86.62 ± 7.30	77.28 ± 7.3	80.66 ± 8.80
Sex M/F	3/5	5/3	10/3	6/3
UPDRS scores			42.16 ± 12.42	40.12 ± 11.83
H&Y stages			3.27 ± 0.95	3.40 ± 0.89
PMI (hours)	6.68 ± 3.76	8.15 ± 4.14	5.73 ± 2.69	5.85 ± 3.98

MMD-LB, mild motor deficits plus Lewy bodies; PD, Parkinson’s disease; PSP, progressive supranuclear palsy; UPDRS, united Parkinson’s disease rating scale, H&Y, Hoehn and Yahr.

### 2.2 Tissue processing and postmortem evaluation

At autopsy, the brains were removed from the calvarium and processed as described previously (Chu et al., 2006). Briefly, each brain was hemisected and then cut into 2 cm coronal slabs. The slabs were fixed in 4% paraformaldehyde for 5 days at 4°C. After 24 brain blocks were sampled from one side of the brain for pathologic diagnoses, the remaining brain slabs were cryoprotected in 0.1 M phosphate buffered saline (PBS; pH 7.4) containing 2% dimethyl sulfoxide and 10% glycerol for 2 days followed by 2% dimethyl sulfoxide and 20% glycerol in PBS for at least 2 days prior to sectioning. The fixed slabs were then cut into 18 adjacent series of 40 μm-thick sections on a freezing sliding microtome. All sections were collected and stored at −20°C in a cryoprotectant solution before processing.

A complete neuropathologic evaluation was performed (Schneider et al., 2006). Dissection of diagnostic blocks was sampled from a hemisected brain, including the substantia nigra and striatum. When present, Lewy bodies were identified with H&E staining and further visualized with antibody staining for α-syn from midfrontal, midtemporal, inferior parietal, anterior cingulate, entorhinal cortex, hippocampus, amygdala, basal ganglia, and midbrain tissue blocks. McKeith criteria (McKeith et al., 2005) were modified to assess the categories of Lewy body disease. Nigral neuronal loss was estimated in the midbrain. For PSP pathologic evaluation, except for neuronal loss, globose neurofibrillary tangles, 4-repeat tau, and tau-positive astrocytes and gliosis were examined in the basal ganglia, cerebellum, and brainstem.

### 2.3 Immunohistochemistry

An immunoperoxidase labeling method was used to visualize microglial and neuronal markers with following antibodies: microglia using monoclonal (1:2,000, Cat# NBP2-7698, Navision) and a polyclonal (1:1,000, Cat# ab185333, Abcam) TMEM119 antibodies, nigral neurons using a polyclonal chemokine C-X3-C motif ligand 1 (CX3CL1; 1:2,000, Cat#10636-RP03, Sino Biological) antibody, tyrosine hydroxylase (TH; 1:10,000, Cat# 22941, ImmunoStar), and CD4^+^ T lymphocytes with a monoclonal CD4 antibody (1:2,000, Cat# ab133616, abcam). Endogenous peroxidase was quenched by a 20 min incubation in 0.1 M sodium periodate, and background staining was blocked by 1 h incubation in a solution containing 2% bovine serum albumin and 5% normal horse serum or goat serum. Tissue sections were incubated with the primary antibody overnight at room temperature. After 6 washes, sections were sequentially incubated for 1 h in biotinylated horse anti-mouse IgG (Cat# BA-2000) or goat anti-rabbit IgG (Cat# BA1000), followed by the *Elite* avidin–biotin complex (1,500; Cat# PK-6100, Vector) for 75 min. The immunohistochemical reaction was completed with 0.05% 3, 3′-diaminobenzidine (DAB) and 0.005% H_2_O_2_. Stained sections were mounted on gelatin-coated slides, dehydrated through graded alcohol, cleared in xylene, and coverslipped with Cytoseal (Richard-Allan Scientific, Cat# 830-16).

Immunohistochemical control experiments included secondary antibodies alone (which control the specificity of the staining procedure). The control sections were processed in a manner identical to that described above. All secondary antibodies alone in control experiments resulted in the absence of specific staining. A pre-adsorption control experiment for the CX3CL1 antibody was also performed. Briefly, the CX3CL1 antibody was combined with a fivefold (by mass) amount of CX3CL1 (10636-Ho8H) recombinant protein in TBS and incubated overnight at 4°C. The immune complexes with the antibody and blocking peptide were centrifuged at 10,000 g for 20 min. The adsorbed peptide/antibody supernatant was then used in lieu of the primary antibody. This resulted in a total absence of staining (Supplementary Figure 1). Additionally, the staining patterns for TMEM119 and CD4 were similar to what has been reported previously (Bennett et al., 2016; Smolders et al., 2018; Olanow et al., 2019; Williams et al., 2020).

### 2.4 Evaluating TMEM119 immunoreactive microglia and CD4^+^ T cells and CX3CL1 and TH immunoreactive neurons

The density of nigral TMEM119-immunopositive (TMEM119-ir) microglial cells, CD4^+^ T cells, CX3CL1-ir, and tyrosine hydroxylase immunoreactive (TH-ir) neurons was estimated for each subject using stereology. All stereological estimates were separately performed using a uniform, systematic, and random design. An optical fractionator unbiased sampling design was used to estimate cell numbers and Cavalieri’s principle was used to estimate the volume within the substantia nigra (Gundersen and Jensen, 1987; Chu et al., 2012). In each subject, we evaluated the substantia nigra pars compacta from the level of the midbrain at the exit of the 3rd nerve to the decussation of the superior cerebellar peduncle. Approximately five equispaced sections were sampled from each brain. The section sampling fraction (ssf) was 1/0.055. The distance between sections was approximately 0.72 mm. In cross-section, the substantia nigra is in the ventral midbrain. The substantia nigra pars compacta was outlined using a 1.25× objective according to the distribution of neuromelanin laden (NM-laden) neurons that are an endogenous marker for dopaminergic neurons. A systematic sample of the area occupied by the substantia nigra pars compacta was made from a random starting point (StereoInvestigator v2021.1.3 software). Counts were made at regular, predetermined intervals (*x* = 313 μm, *y* = 313 μm), and a counting frame (70 × 70 μm = 4,900 μm^2^) was superimposed on images obtained from tissue sections. The area sampling fraction (asf) was 1/0.05. These sections were then analyzed using a 60× Planapo oil immersion objective with a 1.4 numerical aperture. The section’s thickness was empirically determined. Briefly, as the top of the section was first brought into focus, the stage was zeroed at the *z*-axis by the software. The stage then stepped through the *z*-axis until the bottom of the section was in focus. Section thickness averaged 16.24 ± 2.5 μm in the midbrain. The disector height (counting frame thickness) was 10 μm. This method allowed for 1 μm top guard zones and at least 2 μm bottom guard zones. The thickness sampling fraction (tsf) was 1/0.63. Care was taken to ensure that the top and bottom forbidden planes were never included in the cell counting. Immunohistochemistry revealed that CD4^+^ cells were distributed within brain parenchyma and within vascular and perivascular spaces (Supplementary Figure 2). CD4^+^ cells within the vasculature were spread in groups, within perivascular spaces were distributed in a chain shape, and in the parenchyma were individually interspersed. The CD4^+^ cell number in the vascular and perivascular spaces could be influenced by blood flow out from the vasculature during autopsy processes. Therefore, only CD4^+^ cells within the parenchyma were evaluated in the stereological analysis. The ultimate estimate of the counted profiles within the substantia nigra pars compacta was calculated separately using the following formula: *N* = Σ*Q*^−^·1/ssf⋯1/asf⋯1/tsf. Σ*Q*^−^ was the estimated number of raw counts. 1/ssf was section sampling fraction. 1/asf was area sampling fraction. 1/tsf was thickness sampling fraction.

The area estimation of the substantia nigra pars compacta was performed using a 50 × 50 μm point grid with a 10× objective. The total volume of the substantia nigra pars compacta was calculated by Cavalieri estimator software (Gundersen and Jensen, 1987; Chu et al., 2006). The densities of TMEM119-positive microglial cells, CD4^+^ T cells, and CX3CL1-positive neurons were separately estimated using the nigral cellular number from the optical fractionator/substantia nigra volume from the Cavalieri estimator (neuronal number/mm^3^). The coefficients of error (CE) were calculated according to the procedure of Gunderson and colleagues as estimates of precision (West and Gundersen, 1990; Schmitz and Hof, 2000). The CE values were 0.12 ± 0.05 (range 0.10 to 0.15) in PD and PSP and 0.10 ± 0.02 (range 0.08 to 0.12) in MMD-LB and control groups.

### 2.5 Double-label immunofluorescence

A double-label immunofluorescence procedure was employed to examine whether the activated microglia were associated with α-syn or tau accumulation and whether the neuronal and endothelial CX3CL1 levels were differentially affected in the substantia nigra in individuals with PD or PSP. After background staining was blocked for 1 h, sections were incubated in the first primary polyclonal antibody (CX3CL1, 1:1,000) or monoclonal antibody (TMEM119, 1:1,000) overnight separately, followed by biotinylated goat anti-rabbit antibody (BA1000, 1:200, Vector Lab) or biotinylated horse anti-mouse antibody (BA2000, 1:200, Vector Lab) for 1 h, and the *Elite* avidin–biotin complex (1:500; PK-6100, Vector) for 1 h. The immunofluorescent labeling was completed with 1:1,000 fluorescein tyramide (6456, TOCRIS) and 0.005% H_2_O_2_. After blockade for 1 h, the sections were then incubated in the second primary antibodies: phosphorylated α-syn monoclonal antibody (p-S129, 1:1000; ab51253/Abcam), α-syn oligomer-specific monoclonal antibody (Syn05, 1:1000; AS132718, Agrisera), anti-phospho-tau monoclonal antibody (AT8, 1:1,000; MN1020, ThermoFisher), or CD34 (1:10,000; 60180-2-Ig, proteintech) overnight followed by goat anti-rabbit secondary coupled to DyLight 649 (1:200, DI1649, Vector) or horse anti-mouse secondary coupled to DyLight 649 (1:200, DI2649, Vector) for 1 h. The stained sections were mounted on gelatin-coated slides and covered using aqueous mounting medium (N5501, Vector).

All immunofluorescence-labeled slides were imaged with a Nikon A1 Confocal microscope at 20× magnification objective with a 488 and 640 nm excitation source and transparent optics. Twelve to twenty images per case were acquired from sections containing the substantia nigra pars compacta. After the acquisition of an image, the stage was moved to the next sampling site to ensure a completely non-redundant evaluation. To maintain the consistency of the scanned image for each slide, the laser intensity, confocal aperture, photomultiplier voltage, offset, electronic gain, scan speed, image size, filter, and zoom were set for a background level whereby autofluorescence was not visible with a control section. These settings were maintained throughout the entire experiment (Chu et al., 2012). To examine the colocalization of TMEM119/α-syn and TMEM119/tau, optical scanning through the cell’s z-axis was performed at 1-μm thickness, and cells suspected of being double-labeled were confirmed with confocal cross-sectional analysis. The intensity mapping slides ranged from 0 to 4,095; 0 represented a maximum black image, and 4,095 represented a maximum bright image.

### 2.6 Evaluating microglia distribution in the substantia nigra

Microglia are highly dynamic cells that undergo distinct morphological changes during activation, with changes in soma size as well as the length and thickness of microglial processes. To further evaluate the immune reaction, we quantitated the percent area occupied by TMEM119-labeling microglia, including cellular body and processes, in the substantia nigra region using the particle analysis program of Image J (Igathinathane et al., 2008; Chu et al., 2024). The fluorescent images taken from TMEM119-stained nigral sections were adjusted using a threshold with the level at default and scale set to 1.282 pixels/micrometer. The measurement of particle size (micrometer) was set to 1-infinity. The images were calibrated to calculate the area of TMEM119-ir particles per square millimeter (mm^2^) of the substantia nigra.

### 2.7 Measurements of CX3CL1 immunofluorescence intensity in nigral neurons and endothelial cells

Fluorescence intensity measurements were performed according to our previously published procedures (Chu et al., 2012, 2014). To examine CX3CL1 expression in remaining nigral neurons, the CX3CL1-labeled perikarya with or without α-syn inclusions or tau aggregates were identified and outlined separately in different channels. Immunofluorescence intensity was measured within individual CX3CL1-labeled somata. Five equispaced sections of the substantia nigra were sampled and evaluated. Greater than one hundred nigral neurons per case were analyzed. The vascular endothelial cells with CX3CL1 labeling were outlined and measured within individual blood vessels. To account for differences in background staining intensity, five background intensity measurements lacking immunofluorescent profiles were taken from each section. The mean of these five measurements constituted the background intensity, which was then subtracted from the measured intensity of each neuron or blood vessel to provide a final intensity value.

### 2.8 Data analysis

GraphPad Prism 4 software was used for statistical analysis. All data were tested for normal distribution (*p* > 0.1) using the Normality test (Gaussian). Clinical data, neuronal number, particle area, and optical density measurements were expressed as mean ± SD and were compared across groups using one-way ANOVA followed by Tukey’s test. Correlational analysis between measures or morphological data (CX3CL-ir/TMEM119-ir cellular number and CX3CL1 intensity/CD4^+^ cellular number) was performed using Spearman’s rank correlation. The level of significance was set at 0.05 (two-tailed).

### 2.9 Digital illustrations

Conventional light microscopic images were acquired using an Olympus microscope (BX61) attached to a Nikon digital camera (DXM1200) and stored as TIFF files. Confocal images were exported from the Nikon laser-scanning microscope with Nikon A1 software and stored as JPEG files. All figures were prepared using Photoshop 7.0 graphics software. Only minor adjustments of brightness were made.

## 3 Results

### 3.1 Qualitative and quantitative observations of CX3CL1 immunoreactivity in nigral neurons

**Figure 1:**
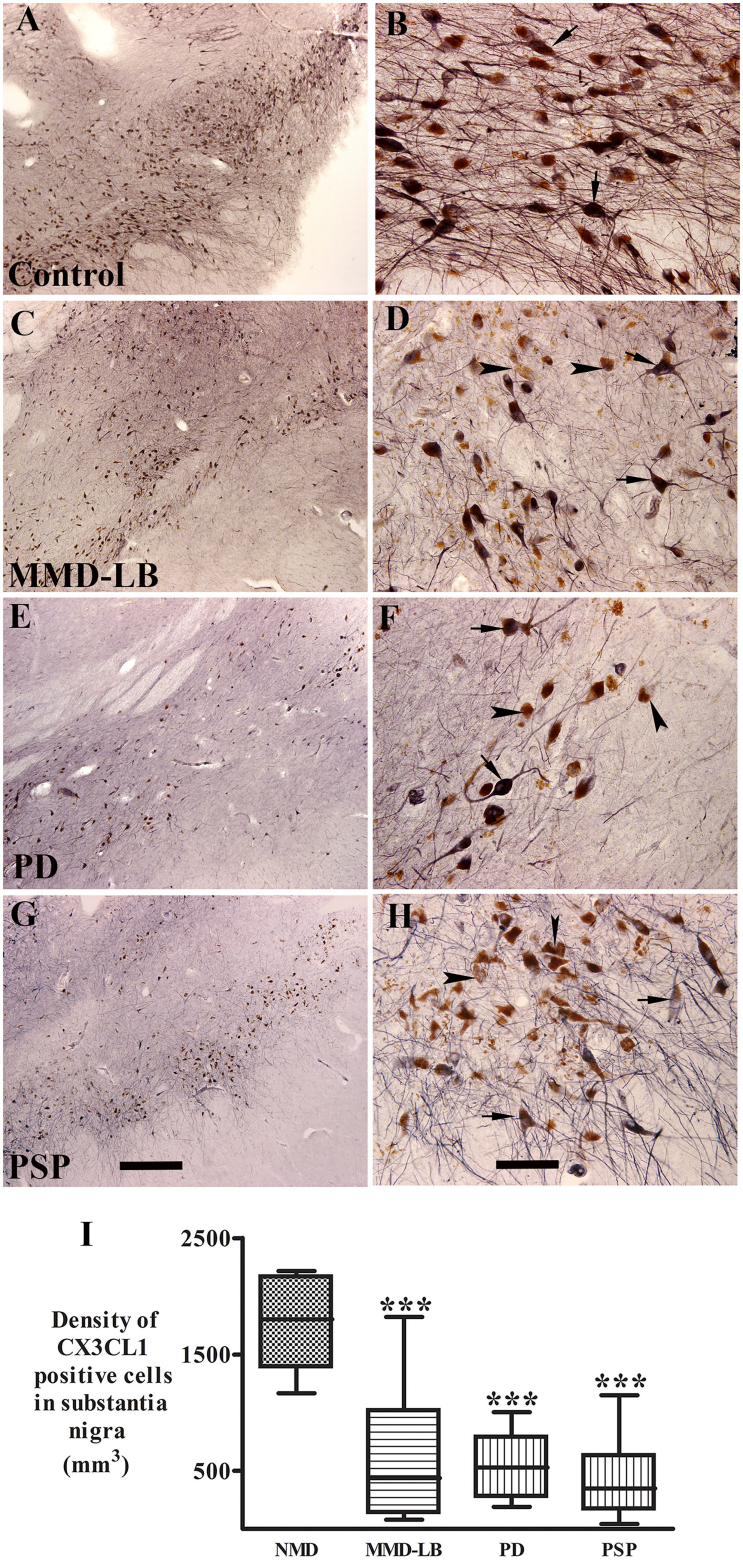
Qualitative and quantitative evaluation of neuronal CX3CL1 immunoreactivity. Photomicrographs of the substantia nigra from control **(A,B)**, MMD-LB **(C,D)**, PD **(E,F)**, and PSP **(G,H)** illustrate CX3CL1 immunoreactivity. Subjects from the control group showed intense CX3CL1-immunoreactive (CX3CL1-ir) somata with extensive local plexuses of processes (arrows; **A,B**) in the nigral neurons. In contrast, neuronal CX3CL1 immunoreactivity was remarkably reduced in subjects with MMD-LB **(C,D)**, PD **(E,F)**, and PSP **(G,H)**. The remaining nigral melanized neurons exhibited light CX3CL1-ir somata with fewer processes (arrows; **D,F,H**) or undetectable CX3CL1 immunoreactive processes (arrowheads; **D,F,H**). Scale bar = 500 μm in **G** (applies to **A,C,E**) and 100 μm in **H** (applies to **B,D,F**). **(I)** Stereological analyses revealed that the density of CX3CL1-positive neurons was significantly reduced in MMD-LB (*n* = 8), PD (*n* = 13), and PSP (*n* = 9) relative to the control group (*n* = 8) (^***^p < 0.001). One-way ANOVA followed by Tukey’s multiple comparison test. Stereological data from five equispaced midbrain sections in each subject. The distance between sections was approximately 0.72 mm. An optical fractionator unbiased sampling design was used to estimate CX3CL1-positive cell numbers and Cavalieri’s principle was used to estimate the volume within the substantia nigra. The densities of CX3CL1-positive neurons were calculated using the estimated CX3CL1-positive cell number from the optical fractionator/substantia nigra volume from the Cavalieri estimator (neuronal number/mm^3^).

To understand whether neuroinflammation is associated with changes in CX3CL1 expression, we examined CX3CL1-immunopositive cells in the substantia nigra in synucleinopathy and tauopathy cases. Morphological analyses revealed that CX3CL1-immunoreactivity was found in perikarya and main processes in both melanized and non-melanized nigral neurons (Figure 1B). In the control group, intense and extensive CX3CL1-immunopositive neurons with abundant processes were widely distributed throughout the substantia nigra (Figure 1A). Most neuromelanin-laden neurons were strongly CX3CL1-immunopositive (Figure 1B). In the MMD-LB group, CX3CL1-immunoreactivity was severely reduced (Figure 1C). Although many NM-laden neurons could be identified, most were CX3CL1-immunonegative (Figure 1D). Some nigral neurons displayed CX3CL1-positive somata with less extensive processes. In the PD group, most NM-laden neurons were CX3CL1-immunonegative (Figures 1E,F), although a few nigral neurons displayed light CX3CL1-immunostaining. Similarly, a majority of the remaining nigral NM-laden neurons were CX3CL1-immunonegative in PSP (Figures 1G,H). To determine whether CX3CL1 was selectively downregulated, we conducted a double labeling of tyrosine hydroxylase (TH) and CX3CL1. Our colocalization analysis of CX3CL1, TH, and NM revealed that NM-laden neurons in the substantia nigra exhibited varying intensities of CX3CL1 and TH staining intensities in disease groups. Specifically, we observed three patterns: neurons with both intensive CX3CL1 and TH staining, neurons that displayed TH staining but lacked CX3CL1 staining, and neurons that showed an absence of both CX3CL1 and TH staining in the disease groups (Supplementary Figure 3).

Stereological analyses revealed that densities of CX3CL1-immunoreactive NM-laden neurons were significantly reduced in MMD-LB (634.54 ± 609.89/mm^3^), PD (487.80 ± 279.20/mm^3^), and PSP (433.51 ± 348.08/mm^3^) groups as compared to controls (1720.84 ± 391.84/mm^3^) (*p* < 0.0001, Figure 1I). *Post hoc* analyses demonstrated that there was a significant difference in MMD-LB (*p* < 0.001), PD (*p* < 0.001), and PSP (*p* < 0.001) relative to controls, but there was no difference among MMD-LB, PD, and PSP groups (*p* > 0.05).

### 3.2 Co-localization and quantitative analyses of CX3CL1 expression in nigral neurons with α-syn or tau inclusions

**Figure 2:**
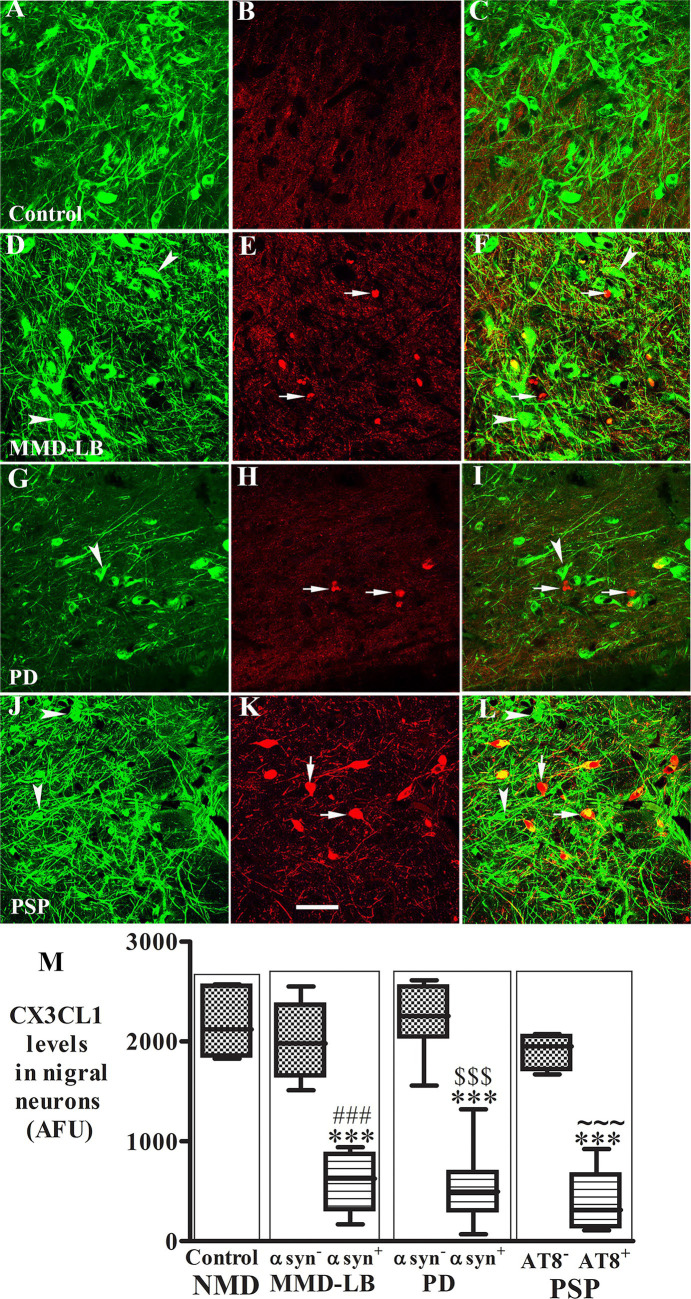
Reduction of CX3CL1 levels in nigral neurons with Syn05-ir or AT8-ir aggregates. Confocal microscopic images of the substantia nigra from control **(A–C)**, MMD-LB **(D–F)**, PD **(G–I)**, and PSP **(J–L)** illustrated immunostaining for CX3CL1 (green; **A,D,G,J**), Syn05 (red; **B,E,H**), AT8 (red; **K**), and co-localization CX3CL1/Syn05 (merged; **C,F,I**) or CX3CL1/AT8 (merged; **L**). CX3CL1 immunofluorescent intensity was extensively reduced in the neurons with α-syn or tau aggregates (arrows; **F,I,L**) but not in neurons without these aggregates (arrowheads; **F,I,L**). Scale bar in **K** = 100 μm (applies to all). Measurements of relative immunofluorescent intensities **(M)** further revealed that CX3CL1 expression was significantly reduced in neurons with Syn05 (αsyn^+^) or tau aggregates (AT8^+^) but not in neurons without Syn05 (αsyn^−^) or tau aggregates (AT8^−^). One-way ANOVA followed by Tukey’s multiple comparison test; ^***^p < 0.001 related to control; ^###^*p* < 0.05 related to Syn05 immunonegative neurons in MMD-LB, ^$$$^*p* < 0.001 related to Syn05 immunonegative neurons in PD, and ^~~~^*p* < 0.001 related to AT8 immunonegative neurons in PSP groups. AFU, arbitrary fluorescence units. CX3CL1 fluorescent intensity measurements from more than 100 neurons with absent α-syn-or tau-aggregates and 50–70 neurons with α-syn-or tau-aggregates in each case. NMD: no motor deficit.

α-syn and tau inclusions play a role in neurodegeneration (Bengoa-Vergniory et al., 2017; Du et al., 2020). Thus, we examined whether the nigral neurons with α-syn or tau aggregates still expressed the CX3CL1. Double labeling CX3CL1/Syn05 or CX3CL1/AT8 was performed in midbrain sections. Immunofluorescence intensity of CX3CL1 was measured from neurons with or without α-syn-inclusions or AT8-aggregates individually. Co-localization studies revealed that the staining intensity of perikaryal CX3CL1-immunoreactivity in neurons without α-syn immunoreactive (α-syn-ir) or AT8 immunoreactive (AT8-ir) aggregates was similar across MMD-LB, PD, and PSP groups (Figures 2D–L) as compared with the control group (Figures 2A–C). In contrast, there was a significant reduction in CX3CL1 expression in nigral neurons with α-syn-ir inclusions in MMD-LB (Figures 2D–F) and PD (Figures 2G–I). Interestingly, CX3CL1 expression was similarly and equally reduced in nigral neurons that contained AT8-immunoreactive inclusions in subjects with PSP (Figures 2J–L) relative to MMD-LB (Figures 2D–F) and PD group (Figures 2G–I). To unequivocally determine whether decreases in levels of CX3CL1 were associated with inclusions, we quantified the relative intensities of CX3CL1-labeling neurons that did or did not contain α-syn-ir or tau-ir aggregates. Quantitatively, there was a statistically significant difference in the intensity of CX3CL1-immunoreactive labeling across these groups (Figure 2M; *p* < 0.0001). Relative to age-matched controls, the fluorescent intensity of CX3CL1 in the neurons with absent α-syn-positive or tau-ir aggregates was reduced to 9.86% in MMD-LB, 0.12% in PD, and 15.19% in PSP groups (*p* > 0.05). In contrast, nigral neurons that contained α-syn-ir or tau-ir aggregates had a similar and significant reduction in CX3CL1 immunofluorescence intensity (66.36% for MMD-LB, 76.18% for PD, and 80.21% for PSP) versus nigral neurons without inclusions (*p* < 0.001).

### 3.3 Qualitative and quantitative observations of CX3CL1 immunoreactivity in nigral endothelial cells

**Figure 3:**
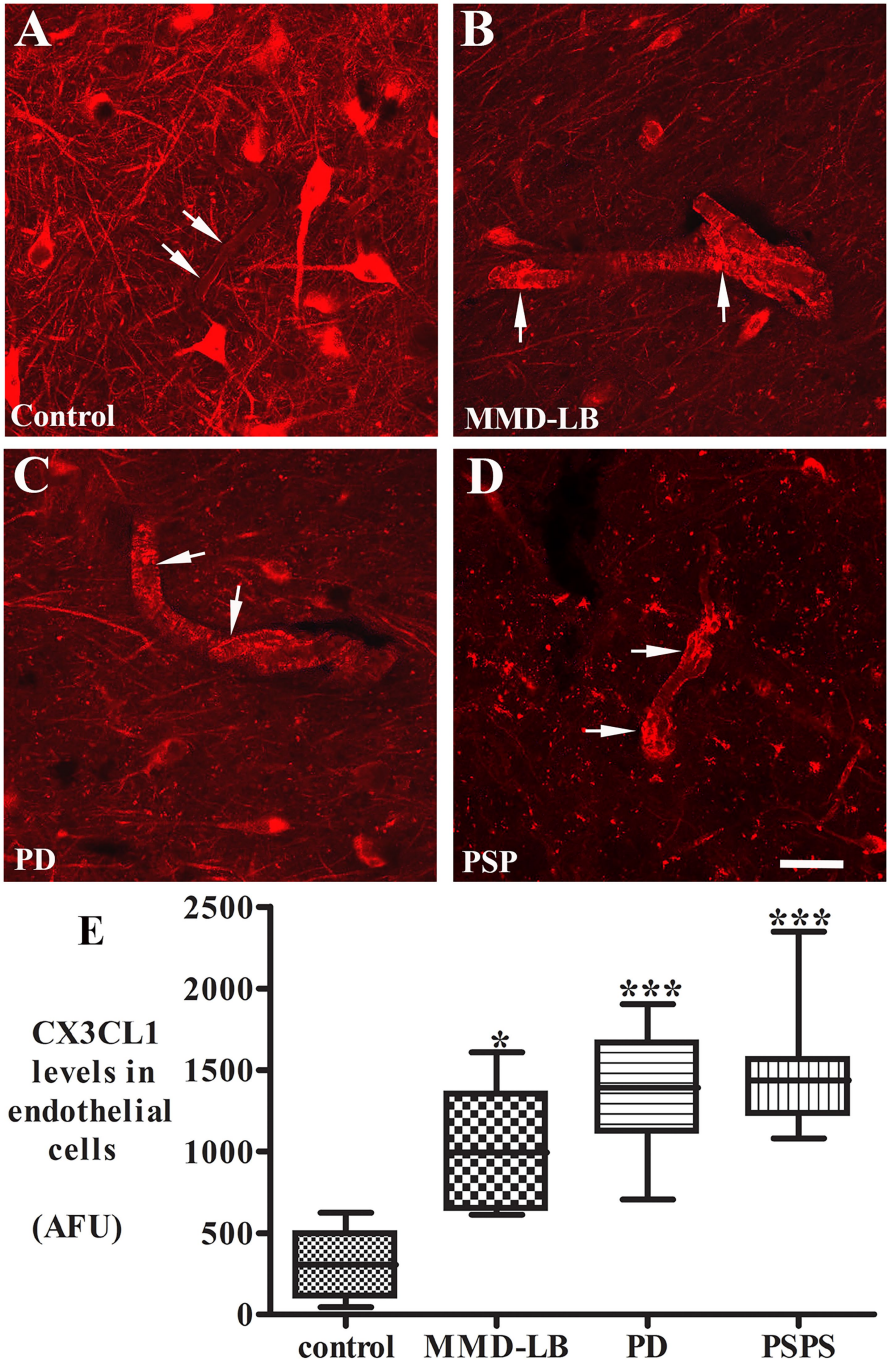
Increased CX3CL1 levels in nigral endothelial cells. Confocal microscopic images of the mid-substantia nigra from control **(A)**, MMD-LB **(B)**, PD **(C)**, and PSP **(D)** CX3CL1 immunoreactivity (arrows) in endothelial cells of blood vessels. Scale bar in **D** = 80 μm (applies to all). Measurement of immunofluorescent intensities **(E)** revealed that CX3CL1 expression significantly increased in endothelial cells in MMD-LB (*n* = 8), PD (*n* = 13), and PSP (*n* = 9) compared with the control (*n* = 8) groups. One-way ANOVA followed by Tukey’s multiple comparison test; ^*^p < 0.05 and ^***^p < 0.001 related to control. CX3CL1 fluorescent intensity measurements from 20–30 blood vessels from five midbrain sections of each case.

CX3CL1 expression in vascular endothelial cells is usually upregulated by inflammatory stimuli such as TNF-α, IL-1, or lipopolysaccharide (Bazan et al., 1997). Whether CX3CL1 expression is changed in nigral endothelial cells with synucleinopathy and tauopathy is unknown. Thus, we examined CX3CL1-immunolabeling in nigral blood vessels. Morphological analyses revealed that CX3CL1 labeled a single cell layer lining the blood vessel (Figure 3B). In the control group, light or undetectable CX3CL1-immunolabeling was observed in vascular endothelial cells (Figure 3A). In contrast, intense CX3CL1-immunostaining was observed on some blood vessels in subjects with MMD-LB, PD, and PSP (Figures 3B–D). To further investigate the location of CX3CL1 expression on blood vessels, a double-labeling technique was employed with CX3CL1 and CD34 antibodies. The CD34 antibody is commonly used to identify and characterize blood vessels, particularly endothelial cells (Fina et al., 1990). This experiment revealed that some CD34-positive blood vessels had intensive CX3CL1 labeling, and others were lower or undetectable in disease groups (Supplementary Figure 4). Lower or undetectable CX3CL1 labeling was observed in the control group. When quantified, the relative intensity of CX3CL1-stained endothelial cells was significantly higher in MMD-LB (996.05 ± 358.01), PD (1350.62 ± 355.70), and PSP (1485.01 ± 389.63) as compared with age-matched control (300.74 ± 221.02) groups (*p* < 0.0001; Figure 3E). *Post hoc* analyses demonstrated that there was a significant difference in MMD-LB (*p* < 0.05), PD (*p* < 0.001), and PSP (*p* < 0.001) as compared with controls, but no differences were seen among MMD-LB, PD, and PSP groups (*p* > 0.05).

### 3.4 Morphologic features and stereological estimates of TMEM119-immunoreactive microglia

**Figure 4:**
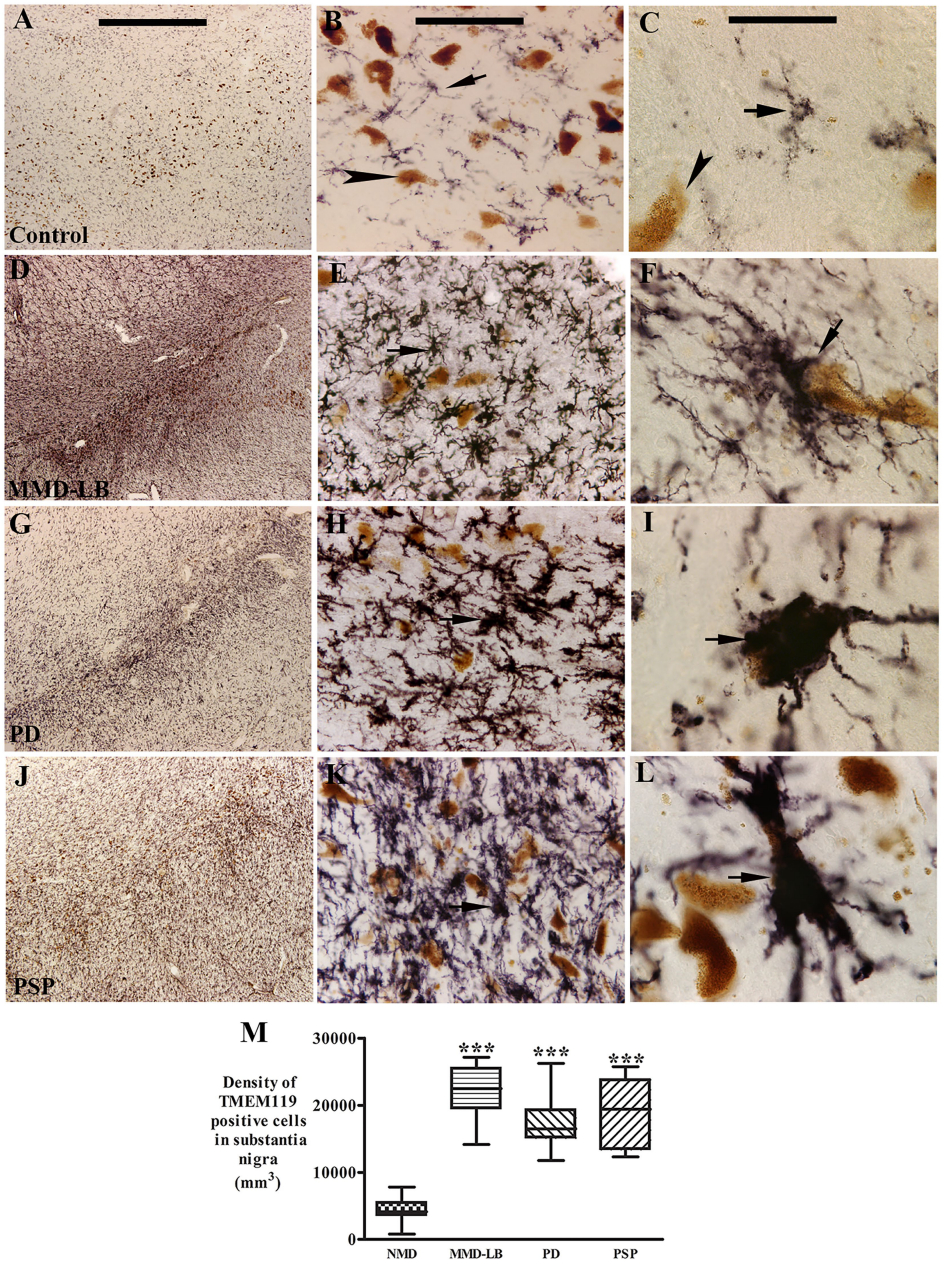
Qualitative and quantitative evaluation of nigral TMEM119 expression. Photomicrographs of the substantia nigra from control **(A–C)**, MMD-LB **(D–F)**, PD **(G–I)**, and PSP **(J–L)** illustrate TMEM119 immunoreactivity. Subject from the control group showed light TMEM119-immunoreactive (TMEM119-ir) intensity **(A,B)** and TMEM119-ir microglia displayed smaller somas with slim ramified processes (arrow, **B,C**) that were distributed among neuromelanin-laden neurons (brown, arrowheads, **B,D**). Intense TMEM119-ir microglia were observed throughout the substantia nigra, especially in pars compacta, in subjects with MMD-LB **(D–F)**, PD **(G–I)**, and PSP **(J–L)**. The TMEM119-ir microglia showed enlarged cellular bodies with intricate processes (arrows, **F,I,L**). Scale bar = 1,000 μm in **A** (applies to **D,G,J**), 100 μm in **B** (applies to **E,H,K**), and 25 μm in **C** (applies to **F,I,L**). **(M)** Stereological analyses revealed that the density of TMEM119-ir microglia was significantly increased in MMD-LB (*n* = 8), PD (*n* = 13), and PSP (*n* = 9) relative to the control (*n* = 8) groups (^***^p < 0.001). One-way ANOVA followed by Tukey’s multiple comparison test. Stereological data from five equispaced midbrain sections in each subject. The distance between sections was approximately 0.72 mm. An optical fractionator unbiased sampling design was used to estimate TMEM119-positive cell numbers and Cavalieri’s principle was used to estimate the volume within the substantia nigra. The densities of TMEM119-positive neurons were calculated using the estimated TMEM119-positive cell number from the optical fractionator/substantia nigra volume from the Cavalieri estimator (cell number/mm^3^).

Several reports demonstrated that innate immune responses are generated by synuclein-and tau-opathies associated with downstream neurodegeneration (Roodveldt et al., 2010; Allen Reish and Standaert, 2015; Laurent et al., 2018). To explore if microglia, the most abundant immune cell in the central nervous system, were activated in brains with MMD-LB, PD, and PSP, we first observed microglial morphological alterations using immunohistochemistry. TMEM119 was selected to recognize the cell surface protein specific to brain-resident microglia (Bennett et al., 2016; Olanow et al., 2019). Immunohistochemistry revealed that TMEM119-positive microglia displayed unusual morphological features across groups. Subjects in the age-matched control group displayed TMEM119-positive microglia with smaller perikarya and slimly ramified processes dispersed between neuromelanin-laden neurons, indicative of resting or homeostatic microglia (Figures 4A–C). In contrast, robust dark and dense TMEM119-labeled microglia with intricate processes indicative of activated microglia accumulated in the substantia nigra of subjects with MMD-LB (Figures 4D–F), PD (Figures 4G–I), and PSP (Figures 4J–L). In these disease groups, TMEM119-labeled microglia had enlarged perikarya with both short and large processes (Figures 4E,I,L) that displayed a bushy-like morphology (Ransohoff and Perry, 2009; Vidal-Itriago et al., 2022). Some TMEM119-positive processes elongated to ramify into, or adjacent to, melanized neurons (Figures 4F,I,L). These TMEM119-positive microglial cells expressed human leukocyte antigen-DR isotype (HLA-DR), a member of the major histocompatibility class II (Supplementary Figure 5).

Stereological analyses revealed that densities of TMEM119-positive microglia in the substantia nigra were significantly higher by 408.62% in MMD-LB (22123.65 ± 4183.91/mm^3^), 296.85% in PD (17261.94 ± 4131.50/mm^3^), and 343.99% in PSP (19312.35 ± 5157.73/mm^3^) groups relative to the control (4349.69 ± 1903.06/mm^3^) group (*p* > 0.0001 across groups; Figure 4M). *Post hoc* analyses further revealed a significant difference in the densities of TMEM119-positive microglia in MMD-LB (*p* < 0.001), PD (*p* < 0.001), and PSP (*p* < 0.001) relative to the control group. Interestingly, there was no difference between disease groups (*p* > 0.05). These qualitative and quantitative observations indicate that the increased number of microglia with enlarged perikarya and processes, as well as microgliosis, suggests that nigral microglia are in an “activated state.” Additionally, some microglia appeared to have invaded melanized neurons, suggesting that microglia were directly scavenging degenerating cells.

### 3.5 Examination of the colocalization of TMEM119 and α-syn or tau aggregate

**Figure 5:**
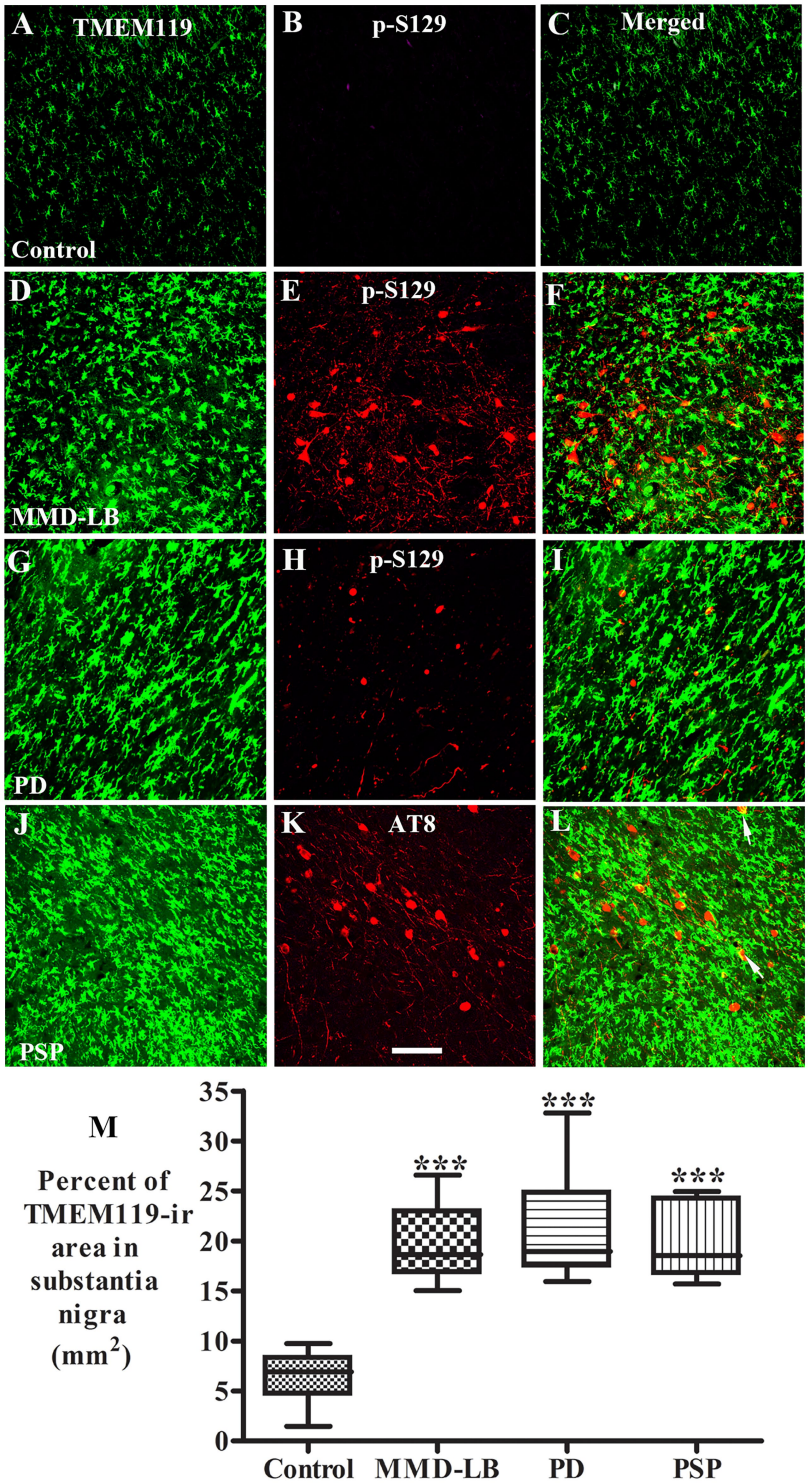
Co-localization analyses of TMEM119 and phosphorylated α-synuclein (p-S129) or phosphorylated tau (AT8). Confocal microscopic images of the substantia nigra from control **(A–C)**, MMD-LB **(D–F)**, PD **(G–I)**, and PSP **(J–L)** illustrated TMEM119 (green; **A,D,G,J**), p-S129 (red; **B,E,H**), AT8 (red; **K**), and colocalization of TMEM119 or p-S129 (merged; **C,F,I**) or AT8 (merged; **L**). Note that TMEM119 immunofluorescent intensity was extensively increased in MMD-LB, PD, and PSP relative to the control. The colocalization of TMEM119 and p-S129 was rarely detected. Few TMEM119-ir microglia attached and colocalized with AT8-labeled inclusions (arrows). Scale bar = 100 μm in **K** (applies to all). **(M)** Measurements of TMEM119-immunoreactive area per square millimeter of the substantia nigra were remarkably increased in MMD-LB (*n* = 7), PD (*n* = 10), and PSP (*n* = 7) compared with the control (*n* = 8) groups (^***^p < 0.001). One-way ANOVA followed by Tukey’s multiple comparison test. Five midbrain sections from each case were measured.

α-syn or tau released from dead or dying neurons can be taken up by microglia, initiating and promoting activation (Dufty et al., 2007; Sulzer et al., 2017; Cascella et al., 2021). Therefore, we performed double labeling of TMEM119 with phosphorylated α-syn (p-S129) or phosphorylated tau (AT8) to examine whether the pathological α-syn or tau was taken up by microglia. Double labeling revealed that microglia surrounded p-S129-positive (Figure 5) or AT8-positive inclusions (Figure 5). However, the colocalization of p-Ser129 and TMEM119 was rare in the substantia nigra (Supplementary Figure 6). Few TMEM119-labeled microglia were co-labeled with AT8-stained inclusions. As α-syn oligomers are known to play a key role in inducing neuroinflammation (Forloni et al., 2016; La Vitola et al., 2018), we used an additional oligomer-specific α-syn (Syn05) antibody to examine whether microglia engulf oligomeric α-syn. Occasionally, TMEM119-positive microglia process apposed α-syn-oligomeric aggregates (Supplementary Figure 7) in subjects with MMD-LB and PD. Cross-sectional analyses further verified that TMEM119/α-syn oligomers were co-localized in some microglia (Supplementary Figures 7A–D). Rarely, TMEM119-labeled microglia co-localized with AT8 in subjects with PSP (Supplementary Figure 8).

As activated microglia undergo rapid morphological transitions in response to stimuli and to adapt to their microenvironment, we further evaluated the TMEM119-immunopositive area in the substantia nigra to assess microglial morphological alterations. Particle analysis (Figure 5M) revealed that the TMEM119-immunofluorescent area was increased by 213.26% in MMD-LB (20.08 ± 3.90/mm^2^), 229.48% in PD (21.12 ± 4.76/mm^2^), and 211.54% in PSP (19.97 ± 3.94/mm^2^) relative to the controls (6.41 ± 2.23/mm^2^). There was a statistically significant difference across these groups (*p* < 0.0001). *Post hoc* analyses demonstrated a significant increase in the nigral TMEM119-immunofluorescent area in subjects with MMD-LB (*p* < 0.001), PD (*p* < 0.001), and PSP (*p* < 0.001) compared with the control group. Critically, there was no difference between disease groups (*p* > 0.05), supporting that synucleinopathy and tauopathy are involved in microglial activation during nigral neurodegeneration.

### 3.6 Qualitative and quantitative observations of CD4^+^ T cell infiltration in the substantia nigra

CD4^+^ T helper cells coordinate immune responses by stimulating other immune cells, such as macrophages, B lymphocytes, and CD8^+^ T lymphocytes, to fight pathogens (Contaldi et al., 2022) and can infiltrate affected brain regions, contributing to neuroinflammation (Sonar and Lal, 2017; Chhatbar and Prinz, 2021). The infiltrated CD4^+^ cells mediate neuroinflammation in PD (Sulzer et al., 2017; Contaldi et al., 2022) and drive disease pathogenesis in animal models (Williams et al., 2021). In this regard, CD4^+^ cells in the substantia nigra were examined and quantified in all subjects. Immunohistochemistry revealed CD4^+^ cells with elongated-flattened, or round-flattened shapes throughout the substantia nigra (Supplementary Figure 2). Some were interspersed in the parenchyma (Supplementary Figures 2B–D), and others gathered in vascular or perivascular spaces (Supplementary Figure 2E). Double labeling of CD4 and CD34 further confirms the presence of CD4^+^ cells in CD34-positive vasculature (Supplementary Figure 9).

The densities of CD4^+^ cells in nigra parenchyma were significantly increased by 970.82% in the MMD-LB (559.08 ± 424.31/mm^3^), 623.09% in PD (377.53 ± 222.80/mm^3^), and 666.55% in PSP (400.22 ± 153.98/mm^3^) groups relative to the control (52.21 ± 33.68/mm^3^) group (*p* > 0.01 across groups; Supplementary Figure 2F). *Post hoc* analyses further revealed a significant difference in the densities of CD4^+^ cells in MMD-LB (*p* < 0.01), PD (*p* < 0.05), and PSP (*p* < 0.05) relative to the control group. There were no differences among the MMD-LB, PD, and PSP groups (*p* > 0.05).

### 3.7 Characteristics of TH expression in substantial nigra

Stereological and particle analyses revealed that microglial and CD4^+^ cells were significantly increased in the MMD-LD, PD, and PSP groups. TH expression in the substantia nigra was examined to determine if this increase in inflammation was occurring alongside dopaminergic neurodegeneration, as dopaminergic neurodegeneration is associated with inflammation (Pajares et al., 2020; Harms et al., 2023). Subjects from the control group had a high density of TH immunoreactive (TH-ir) somata with an intricate plexus of TH-ir processes (Supplementary Figures 10A,B). MMD-LB subjects displayed an obvious reduction in TH immunoreactivity (Supplementary Figures 10C,D) compared with the control (Supplementary Figures 10A,B). TH-ir neurons showed less extensive processes, and many neuromelanin-laden (NM-laden) neurons were TH-immunonegative (Supplementary Figure 10D). As expected, in PD subjects, both TH-ir somata and dendrites in the substantia nigra (Supplementary Figures 10E,F) were severely reduced to a degree greater than that seen in subjects with age-matched controls and MMD-LB. Similarly to PD, subjects from the PSP group showed severely reduced TH immunoreactivity, and many NM-laden neurons were TH immunonegative (Supplementary Figure 10H).

Stereological analyses confirmed that the density of TH-ir neurons in MMD-LB subjects (1327.20 ± 469.32/mm^3^) was decreased between age-matched controls (2277.81 ± 232.27/mm^3^) and PD (532.95 ± 256.54). The density of TH-ir neurons in PSP subjects (725.89 ± 574.45) was similar to PD group. Relative to the age-matched control group, the reduction of nigral TH-ir neurons was 54.22% in MMD-LB, 81.61% in PD, and 80.18% in PSP (*p* > 0.01 across groups; Supplementary Figure 8I). Post hoc analyses demonstrated a significant reduction of TH-ir neurons in MMD-LB (*p* < 0.001), PD (*p* < 0.001), and PSP (*p* < 0.001) compared with the age-matched control group and between the MMD-LB and PD groups (*p* < 0.01).

### 3.8 Correlative analyses of CX3CL1, TMEM119, and CD4 immunoreactivities

**Figure 6:**
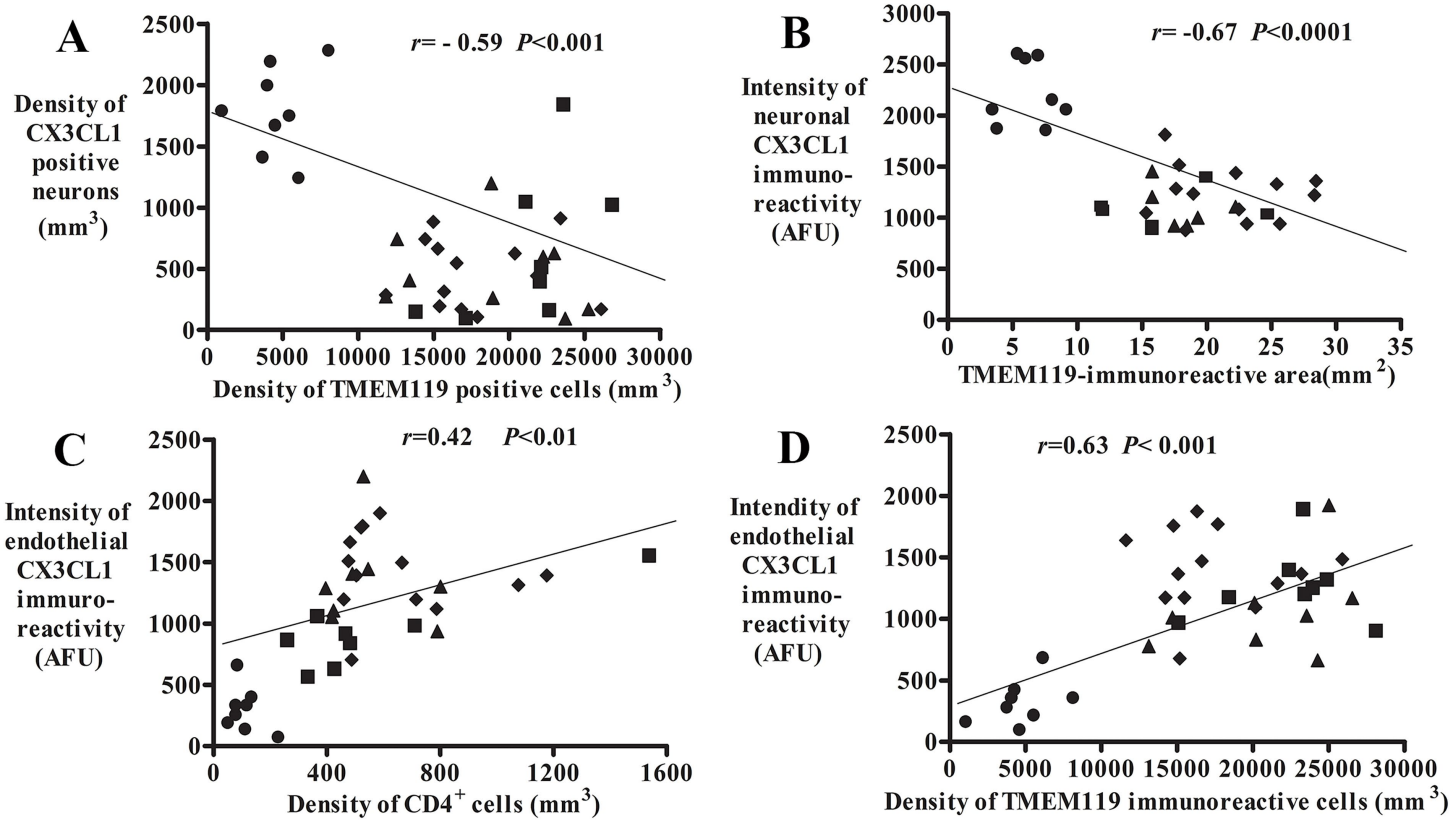
Correlative analyses of CX3CL1, TMEM119, and CD4 immunoreactivities. Scatterplots show the correlation between the density of CX3CL1-ir neurons/TMEM119-ir microglial cells (**A**, *n* = 38), neuronal CX3CL1 intensity/TMEM119-immunoreactive area (**B**, *n* = 32; 1 MMD-LD, 3 PDs, and 2 PSP cases absent TMEM119-immunoreactive area), endothelial CX3CL1-ir intensity/CD4^+^ cellular density (**C**, *n* = 36; 2 PSP cases absent CD4 cell number), and endothelial CX3CL1 intensity/TMEM119-ir microglial density (**D**, *n* = 38). ● Control, ■ MMD-LB, ◆ PD, and ▲ PSP.

A regression analysis demonstrated that there was a negative correlation between the densities of CX3CL1-ir neurons and TMEM119-ir microglial numbers (*r* = −0.59; *p* < 0.001; Figure 6A) as well as neuronal CX3CL1-ir fluorescence intensity and TMEM119-ir microglial area (*r* = −0.67; *p* < 0.0001; Figure 6B). The intensity of endothelial CX3CL1 immunoreactivity were positively correlated with the density of infiltrated CD4^+^ cellular number (*r* = 0.42; *p* < 0.05; Figure 6C) and the density of TMEM119-ir microglial cells (*r* = 0.63; *p* < 0.001; Figure 6D) in the substantia nigra. Except for lower correlations (*r* values) between age vs. CX3CL1-ir neuronal number and UPDRS vs. TMEM119-ir microglial area, there was no correlation of morphological data of CX3CL1 and TMEM119 vs. age, united Parkinson’s disease rating scale (UPDRS), and Hoehn and Yahr (H&Y) (Supplementary Table S1).

## 4 Discussion

The present study investigates neurons and endothelial cells expressing CX3CL1, microglia expressing TMEM119, and CD4-expressing cells in subjects with MMD-LB, PD, PSP, and age-matched controls. The results from immunohistochemical examination of postmortem brains revealed that CX3CL1-expressing neuronal numbers and levels were relatively reduced, and CX3CL1 expression in vascular endothelial cells was augmented, nigral microglial and CD4^+^ cells were relatively increased in MMD-LB and PD subjects displaying synucleinopathy. The alterations of CX3CL1, TMEM119, and CD4 in subjects with tauopathy also displayed a similar pattern of inflammatory changes. The increase in microglial number was inversely correlated with the reduced number of CX3CL1-expressing neurons. The increases in nigral microglial and CD4^+^ cell numbers were associated with the increase in CX3CL1 expression in endothelial cells. These data suggest that inverse expression of CX3CL1 from nigral neurons and endothelial cells contributes to microglial activation and CD4^+^ cell infiltration during disease progression.

Downregulation of CX3CL1 expression was observed within remaining nigral neurons in PD postmortem tissue. It is unclear whether the CX3CL1 expression downregulation exists merely in advanced PD, as many neurons are lost in advanced PD. To illustrate this alteration during disease development, we examined the brains from subjects with mild motor deficits but with clinical syndromes insufficient for a PD diagnosis. These brains had a greater number of dopaminergic neurons and contained nigral Lewy bodies and neurites. Present analyses of nigral TH-ir neurons reconfirmed that the nigral neuronal loss in the MMD-LB group was intermediate between age-matched control and PD groups (Chu et al., 2018, 2024). We have termed this group mild motor deficits plus Lewy body (MMD-LB), and the details of the designation of subjects into this category have been published previously (Chu et al., 2024). In this group, we observed a severe reduction in CX3CL1 immunopositive neurons as well as a reduction in CX3CL1 immunofluorescence intensity, which was similar to what was seen in the PD group, indicating that downregulation of CX3CL1 is an early event. Interestingly, the increase of TMEM119-positive microglia and CD4^+^ cells in the MMD-LB group was similar to the PD group, suggesting that downregulation of CX3CL1 expression is a greater factor than cell loss in driving nigral neuroinflammation. As PD and MMD-LB are synucleinopathies, whether CX3CL1 downregulation also occurs in the brains with tauopathies was unclear. To investigate this question further, we examined clinically and pathologically diagnosed PSP subjects to determine whether the reduction of CX3CL1 expression also occurs in this related disease. Like PD and MMD-LB, a severe reduction of CX3CL1-ir neuronal number and immunofluorescence intensity was observed in the remaining nigral neurons in PSP. Therefore, the decline of neuronal CX3CL1 expression is not specific to synucleinopathies but represents a general inflammatory response. Reduction of neuronal CX3CL1 expression may be a potential mechanism for neuroinflammation.

Our present study showed that the localization of α-syn within microglia was exceedingly rare. The internalization of α-syn by resting and activating microglia may be transient and hard to catch by a one-time morphological assessment in postmortem tissue. We also found that neuronal CX3CL1 expression was significantly decreased, and microglial cells were in an active state in subjects with preclinical and clinical PD. A decrease in CX3CL1 neuronal number was inversely correlated with an increase in the microglial number, indicating that the loss of CX3CL1 expression would result in microglial hyperplasia.

CX3CL1 is constitutively and abundantly expressed in neurons in the central nervous system (Bazan et al., 1997). At normal levels of CX3CL1, microglia regulate development, maintain neuronal networks, and repair injury to create a stable microenvironment for brain health (Colonna and Butovsky, 2017; Umpierre and Wu, 2021). Under pathological conditions, such as PD, many remaining NM-laden neurons lose the ability to express CX3CL1. A lower neuronal CX3CL1 level in the substantia nigra cannot limit CX3CR1 activation and thus the microglial cells are working in an uncontrolled fashion (Wendimu and Hooks, 2022). Activated microglia can release free radicals and proteases to damage neurons, engulf stressed neurons (Brown and Vilalta, 2015), and produce chemokines that recruit white blood cells from the periphery, specifically T cells (Williams et al., 2021; Shi et al., 2023). A series of animal studies supports that CX3CL1 plays a key role in neuroinflammation, as microglia are seen to be activated by the loss of CX3CL1, triggering an inflammatory response that leads to chronic neuroinflammation and neuronal damage (Cardona et al., 2006; Pabon et al., 2011). Conversely, exogenous CX3CL1 can suppress this microglia activation and protect neurons in a toxic PD model (Staniland et al., 2010; Pabon et al., 2011; Mattison et al., 2013). Overexpression of soluble CX3CL1 from the fractalkine gene cloned into the pTR2-MCS vector suppresses α-syn-mediated neurodegeneration and alters the microglial state to a neuroprotective one (Nash et al., 2015). These data from us and others demonstrate that the downregulation of CX3CL1 expression from injured neurons influences the CX3CL1/CX3CR1 signaling axis and neuron–microglia communication, thus leading microglia to become pro-inflammatory.

Conversely, the CX3CL1 expression in endothelial cells was significantly increased in subjects with synucleinopathy or tauopathy. Several reports demonstrate that proinflammatory cytokines can induce endothelial cells to express CX3CL1 (Garcia et al., 2000; Schulz et al., 2007; Hirono et al., 2020). The levels of these cytokines, such as TNF-α and IL-1, are high in the brain, CSF, and blood of PD patients (Tansey and Romero-Ramos, 2018; Pajares et al., 2020). Endothelial cells can receive and relay cytokine signals between the blood and brain (Chen et al., 2020). The proinflammatory cytokines, such as IL-1 or TNFα produced by activated microglia and astroglia, impact endothelial cell functions (Wu et al., 2017). Our study demonstrated that some endothelial cells express more CX3CL1. The chemokine domain acts as a chemoattractant for cells that express CX3CR1, and the mucin-like stalk acts as an adhesion molecule to bind leukocytes (Pawelec et al., 2020). Using chemokines and adhesion molecules, endothelial cells recruit circulating white blood cells into the brain vasculature. Several white blood cells, including monocytes and lymphocytes, express the CX3CR1 receptor (Batista et al., 2020). The CX3CL1 expressed by endothelial cells binds to CX3CR1 and helps monocytes and lymphocytes enter the brain parenchyma (Imai et al., 1997). The present study revealed that CD4^+^ cells accumulated within nigral blood vessels and infiltrated the parenchyma of the substantia nigra in individuals affected by synucleinopathy or tauopathy. Further study is needed to determine whether CX3CR1-positive CD4^+^ cells accumulate within blood vessels and infiltrate the parenchyma, which is associated with upregulated CX3CL1 expression in endothelial cells.

CX3CR1 was not analyzed in this study due to the limitations of existing antibodies. Instead, we analyzed the morphological alteration of microglia using a TMEM119 antibody. The TMEM119 antibody recognizes the intracellular domain of TMEM119 and may enable the differentiation of CNS-resident microglia and blood-infiltrating macrophages (Bennett et al., 2016; Ruan and Elyaman, 2022). We only analyzed CD4^+^ cell distribution in the substantia nigra, though there may be alterations in other T cells such as CD8^+^ cells. CD4 T cells are MHC-II restricted and pre-programmed for helper functions, while CD8 T cells are MHC I-restricted and pre-programmed for cytotoxic function (Xiong and Bosselut, 2012). The CD4^+^ cells were selected for analysis, as microglia express MHC-II during neurodegenerative diseases (Schetters et al., 2018). The infiltrated CD4^+^ cells may regulate microglia activation through MHC-II. Of course, other T cells play an important role in neuroinflammation, and further study is needed.

## 5 Conclusion

The present study demonstrated inverse changes in the expression of CX3CL1 in nigral neurons and endothelial cells. CX3CL1 expression is decreased in neurons while endothelial CX3CL1 expression is upregulated in the substantia nigra of subjects with a synucleinopathy or tauopathy. The reduction of neuronal CX3CL1 may be associated with an increase in microglial hyperplasia while the increase in endothelial CX3CL1 may be related to CD4^+^ cell infiltration in the substantia nigra. These data demonstrate that differing expression of neuronal and endothelial fractalkine in the substantia nigra contributes to neuroinflammatory activity in both synucleinopathies and tauopathy. Due to limitations in neuropathological studies, we cannot determine if the correlation we observed is cause or an effect of neuroinflammation. Further studies should focus on the correlation of neuronal CX3CL1 levels with microglial CX3CR1 alterations to further elucidate the imbalance of these molecules in neuroinflammatory activity. The factors that upregulate neuronal CX3CL1 or downregulate endothelial CX3CL1 levels may offer a novel therapeutic strategy for reducing neuroinflammation in neurodegenerative disease.

## Data Availability

The original contributions presented in the study are included in the article/Supplementary material, further inquiries can be directed to the corresponding author.

## Supplementary material

SUPPLEMENTARY FIGURE 1 Pre-adsorption of CX3CL1 antibodies with a peptide. CX3CL1-immunopositive neurons **(A)** were observed in the substantia nigra without pre-adsorption in the control brain. After pre-adsorption of the CX3CL1 antibody/CX3CL1 peptide, the CX3CL1 staining was undetectable **(B)**. Scale bar in **B** = 100 μm (applies to **A**).

SUPPLEMENTARY FIGURE 2 Qualitative and quantitative evaluation of CD4^+^ cells in the substantia nigra. Photomicrographs of the mid-substantia nigra from control **(A)**, MMD-LB **(B)**, PD **(C,E)**, and PSP **(D)** show CD4^+^ cell distribution. Brown coloring indicates neuromelanin-laden dopaminergic neurons (curved arrows). Arrows denote CD4^+^ T cells in nigral parenchyma and arrowheads denote CD4^+^ cells within blood vessels. Scale bar in **E** = 40 μm (applies to all). Unbiased stereological cell counts **(F)** indicate a significant increase in the number of CD4^+^ T cells in nigral parenchyma. Data: One-way ANOVA followed by Tukey’s multiple comparison test; ^*^*p* < 0.05 and ^**^*p* < 0.01 compared with the control group. Stereological data from five equispaced midbrain sections in age-matched control (*n* = 8), MMD-LB (*n* = 8), PD (*n* = 13), and PSP (*n* = 7). The distance between sections was approximately 0.72 mm. An optical fractionator unbiased sampling design was used to estimate CD4-positive cell numbers and Cavalieri’s principle was used to estimate the volume within the substantia nigra. The densities of CD4-positive cells were calculated using the estimated CD4-positive cell number from the optical fractionator/substantia nigra volume from the Cavalieri estimator (cell number/mm^3^).

SUPPLEMENTARY FIGURE 3 Co-localization analyses of CX3CL1 and TH in the substantia nigra. Confocal microscopic images of the substantia nigra from age-matched control (control; **A–D**), MMD-LB **(E–H)**, PD **(I–L)**, and PSP **(M–P)** brains illustrated CX3CL1 (green; **A,E,I,M**), TH (red; **B,F,J,N**), neuromelanin (NM, black; **C,G,K,O**), and merged CX3CL1 and TH **(D,H,L,P)** immunofluorescent staining. Arrows denote NM-laden neurons with intensive CX3CL1 and TH double labeling (yellow; **D,H,L,P**). Curved arrows indicate NM-laden neurons with TH staining and absent CX3CL1. Arrowheads denote NM-laden neurons absent CX3CL1 and TH labeling. Scale bar in **P** = 100 μm (applies to all).

SUPPLEMENTARY FIGURE 4 Co-localization analyses of CX3CL1 and CD34 in the substantia nigra. Confocal microscopic images of the substantia nigra from age-matched control (control; **A–C**), MMD-LB **(D–F)**, PD **(G–I)**, and PSP **(J–L)** brains illustrated CX3CL1 (green; **A,D,G,J**), CD34 (red; **B,E,H,K**), and merged CX3CL1 and CD34 **(C,F,I,L)** immunofluorescent staining. Arrows denote blood vessels exhibiting intensive CX3CL1 and CD34 labeling (yellow; **C,F,I,L**). Arrowheads denote blood vessels displaying only CD34 labeling (red; **C,F,I,L**). Scale bar in **L** = 100 μm (applies to all).

SUPPLEMENTARY FIGURE 5 Co-localization analyses of TMEM119 and HLA-DR in the substantia nigra. Confocal microscopic images of the substantia nigra from PD brain illustrated TMEM119 (green; **A**), HLA-DR (red; **B**), and merged TMEM119 and HLA-DR **(C)** immunofluorescent staining. Arrows denote enlarged microglial cell with both extensive TMEM119 and HLA-DR staining (yellow; **C**). Arrowheads denote HLA-DR single labeling (red; **B,C**). A cross-section of confocal images further illustrated the colocalization of labeled TMEM119 and HLA-DR **(D)**: the large panel represents a cross-section of the cell layer. The horizontal and vertical lines through them denote the planes of the adjoining *xz* and *yz* sections, respectively. At the bottom and right, the *xz* and *yz* cross-sections were obtained from the combined serial optical sections of these cell layers using Nikon A1 software. The cross-section analyses revealed that TMEM119 was colocalized with HLA-DR. Scale bar in **C** = 100 μm (applies to all).

SUPPLEMENTARY FIGURE 6 Co-localization analyses of TMEM119 and α-synuclein (p-S129) in the substantia nigra. Confocal microscopic images of the substantia nigra from MMD-LB brain illustrated TMEM119 (green; **A**), p-S129 (arrow, red; **B**), and merged TMEM119 and p-S129 **(C)** immunofluorescent staining. Arrows denote p-S129 inclusion. A cross-section of confocal images further illustrated the colocalization of labeled TMEM119 and p-S129 **(D)**: the large panel represents a cross-section of the cell layer. The horizontal and vertical lines through them denote the planes of the adjoining *xz* and *yz* sections, respectively. At the bottom and right, the *xz* and *yz* cross-sections were obtained from the combined serial optical sections of these cell layers using Nikon A1 software. The cross-section analyses revealed that TMEM119 was not colocalized with p-S129. Scale bar in **C** = 100 μm (applies to all).

SUPPLEMENTARY FIGURE 7 Co-localization analyses of TMEM119 and α-synuclein (Syn05) in the substantia nigra. Confocal microscopic images of the substantia nigra from PD brain illustrated TMEM119 (green; **A**), Syn05 (red; **B**), and merged TMEM119 and Syn05 **(C)** immunofluorescent staining. Arrows denote the colocalization of TMEM119 and Syn05 (yellow; **C**). A cross-section of confocal images further illustrated the colocalization of labeled TMEM119 and Syn05 **(D)**: the large panel represents a cross-section of the cell layer. The horizontal and vertical lines through them denote the planes of the adjoining *xz* and *yz* sections, respectively. At the bottom and right, the *xz* and *yz* cross-sections were obtained from the combined serial optical sections of these cell layers using Nikon Confocal software. The cross-section analyses revealed that a few TMEM119 were colocalized with Syn05 (**D**; yellow). Scale bar in **C** = 100 μm (applies to all).

SUPPLEMENTARY FIGURE 8 Co-localization analyses of TMEM119 and tau (AT8) in the substantia nigra. Confocal microscopic images of the substantia nigra from PSP brain illustrated TMEM119 (green; **A**), AT8 (red; **B**), and merged TMEM119 and AT8 **(C)** immunofluorescent staining. Arrows denote colocalization of TMEM119 and AT8. A cross-section of the confocal image further illustrated the colocalization of labeled TMEM119 and AT8 **(D)**: the large panel represents a cross-section of the cell layer. The horizontal and vertical lines through them denote the planes of the adjoining *xz* and *yz* sections, respectively. At the bottom and right, the *xz* and *yz* cross-sections were obtained from the combined serial optical sections of these cell layers using Nikon Confocal software. The cross-section analyses revealed that TMEM119-ir microglia attached to tau inclusions (**D**; yellow). Scale bar in **C** = 100 μm (applies to all).

SUPPLEMENTARY FIGURE 9 Co-localization analyses of CD4 and CD34 in the substantia nigra. Confocal microscopic images of the substantia nigra from PD **(A–C)** and PSP **(D–F)** brains illustrated CD4 (green; **A,D**), CD34 (red; **B,E**), and merged CD4 and CD34 **(C)** immunofluorescent staining. Arrows denote CD4^+^ cells within blood vessels, and the arrowhead denotes CD4^+^ cells within perivascular regions. Scale bar in **E** = 100 μm (applies to all).

SUPPLEMENTARY FIGURE 10 Qualitative and quantitative evaluation of nigral TH expressions. Photomicrographs of the substantia nigra from age-matched control (control; **A,B**), MMD-LB **(C,D)**, PD **(E,F)**, and PSP **(G,H)** brains illustrate TH immunoreactivity. Intense TH-ir soma with an extensive local plexus of TH-ir processes was distributed throughout the substantia nigra **(A,B)** in the control group. Nigral TH immunoreactivity, including somata and processes, was severely reduced in disease groups **(C–H)** compared with control **(A,B)**, and some remaining melanized neurons exhibited no detectable TH immunoreactivity (arrows, brown; **D,F,H**). Scale bar = 500 μm in **G** (applies to **A,C,E**) and 100 μm in **H** (applies to **B,D,F**). **(I)** Stereological analyses revealed that the density of TH-ir neurons was significantly reduced in the MMD-LB (*n* = 8), PD (*n* = 13), and PSP (*n* = 9) groups relative to the control (*n* = 8) groups. One-way ANOVA followed by Tukey’s multiple comparison test; ^***^*p* < 0.001 compared with control; ^##^*p* < 0.01 compared with MMD-LB. Stereological data from five equispaced midbrain sections in each subject. The distance between sections was approximately 0.72 mm. An optical fractionator unbiased sampling design was used to estimate TH-positive cell numbers and Cavalieri’s principle was used to estimate the volume within the substantia nigra. The densities of TH-positive neurons were calculated using the estimated TH-positive cell number from the optical fractionator/substantia nigra volume from the Cavalieri estimator (neuronal number/mm^3^).
