# Supplementary figures and images for: Decreased neuronal and increased endothelial fractalkine expression are associated with neuroinflammation in Parkinson’s disease and related disorders
Source: Front Cell Neurosci. 2025 Aug 6;19:1557645. doi: 10.3389/fncel.2025.1557645 (PMC12364955; doi:10.3389/fncel.2025.1557645)

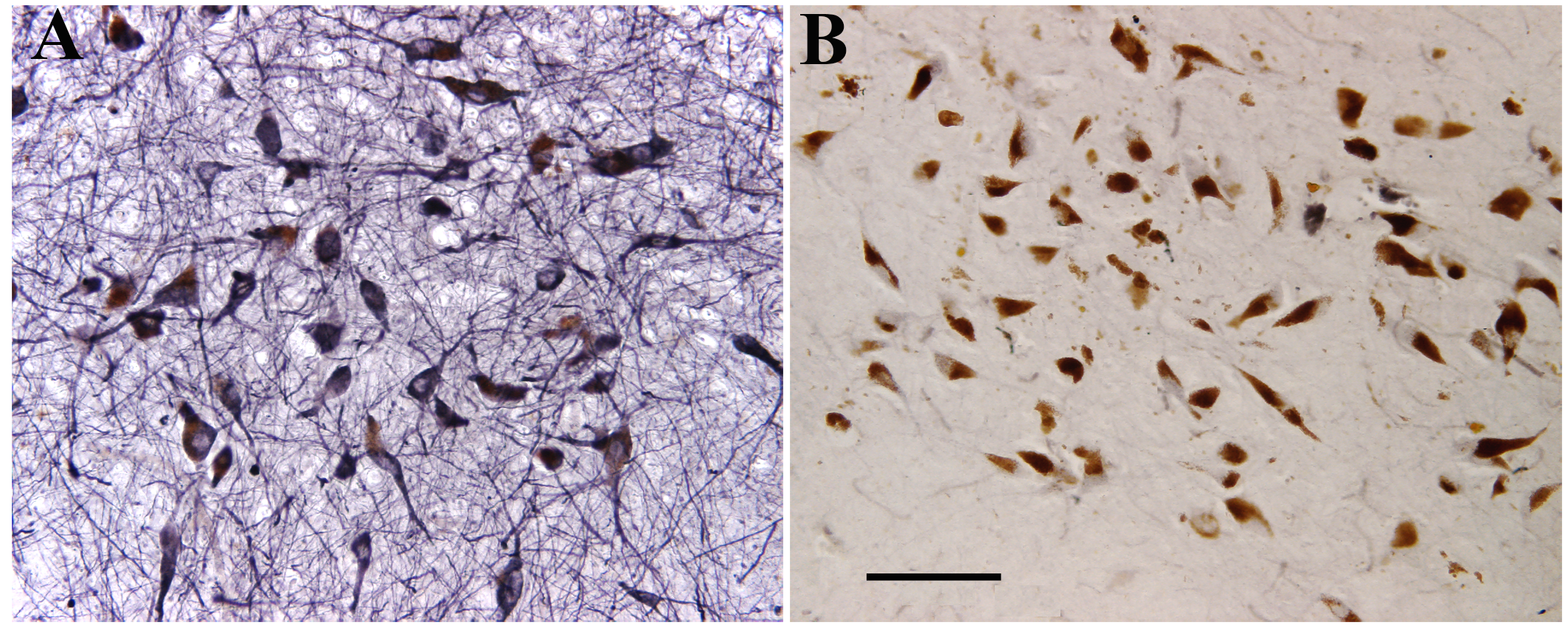

Supplement: SUPPLEMENTARY FIGURE 1 — Pre-adsorption of CX3CL1 antibodies with a peptide. CX3CL1-immunopositive neurons (A) were observed in the substantia nigra without pre-adsorption in the control brain. After pre-adsorption of the CX3CL1 antibody/CX3CL1 peptide, the CX3CL1 staining was undetectable (B). Scale bar in B = 100 μm (applies to A). [file Image_1.tif]

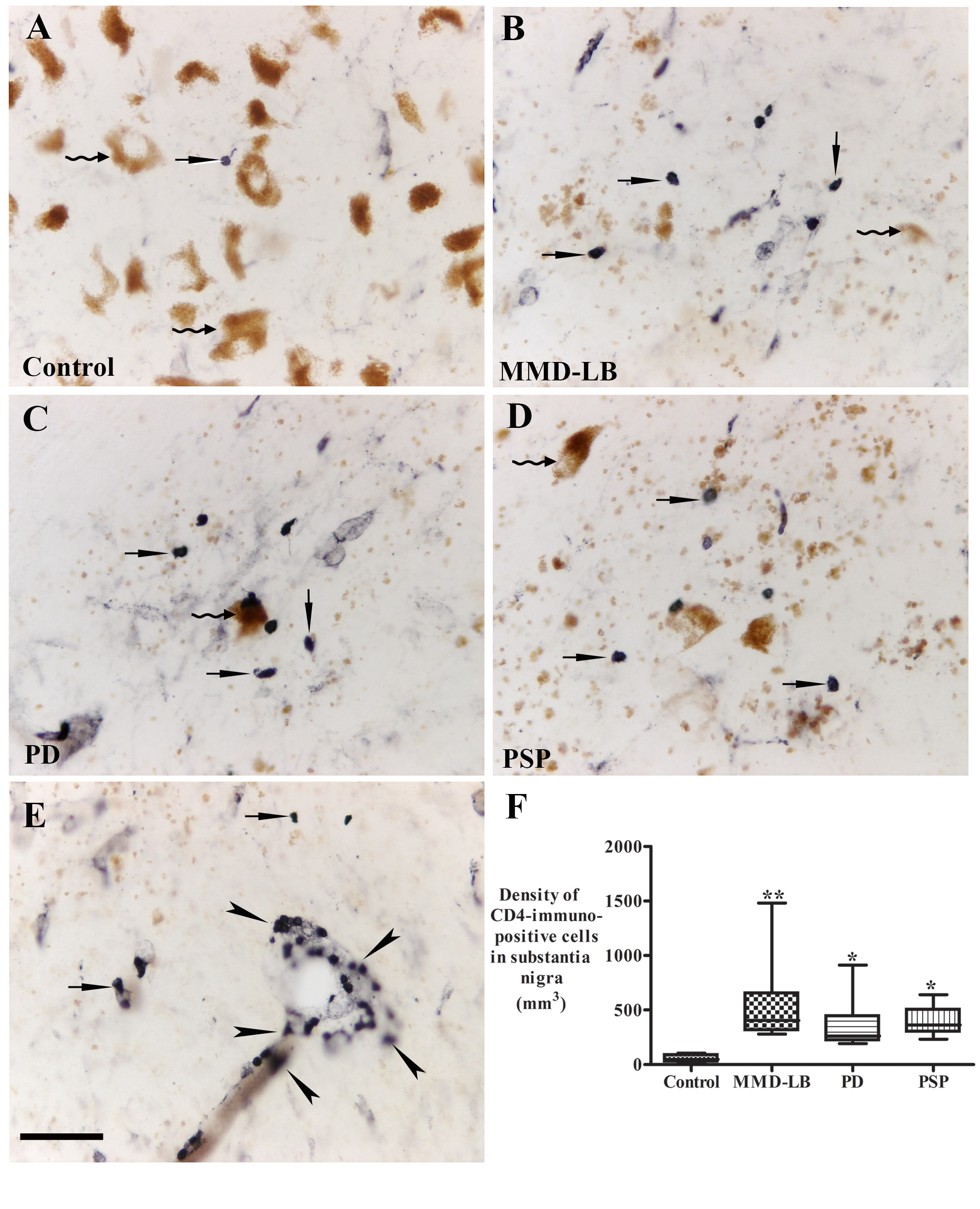

Supplement: SUPPLEMENTARY FIGURE 2 — Qualitative and quantitative evaluation of CD4+ cells in the substantia nigra. Photomicrographs of the mid-substantia nigra from control (A), MMD-LB (B), PD (C,E), and PSP (D) show CD4+ cell distribution. Brown coloring indicates neuromelanin-laden dopaminergic neurons (curved arrows). Arrows denote CD4+ T cells in nigral parenchyma and arrowheads denote CD4+ cells within blood vessels. Scale bar in E = 40 μm (applies to all). Unbiased stereological cell counts (F) indicate a significant increase in the number of CD4+ T cells in nigral parenchyma. Data: One-way ANOVA followed by Tukey’s multiple comparison test; *p < 0.05 and **p < 0.01 compared with the control group. Stereological data from five equispaced midbrain sections in age-matched control (n = 8), MMD-LB (n = 8), PD (n = 13), and PSP (n = 7). The distance between sections was approximately 0.72 mm. An optical fractionator unbiased sampling design was used to estimate CD4-positive cell numbers and Cavalieri’s principle was used to estimate the volume within the substantia nigra. The densities of CD4-positive cells were calculated using the estimated CD4-positive cell number from the optical fractionator/substantia nigra volume from the Cavalieri estimator (cell number/mm3). [file Image_2.tif]

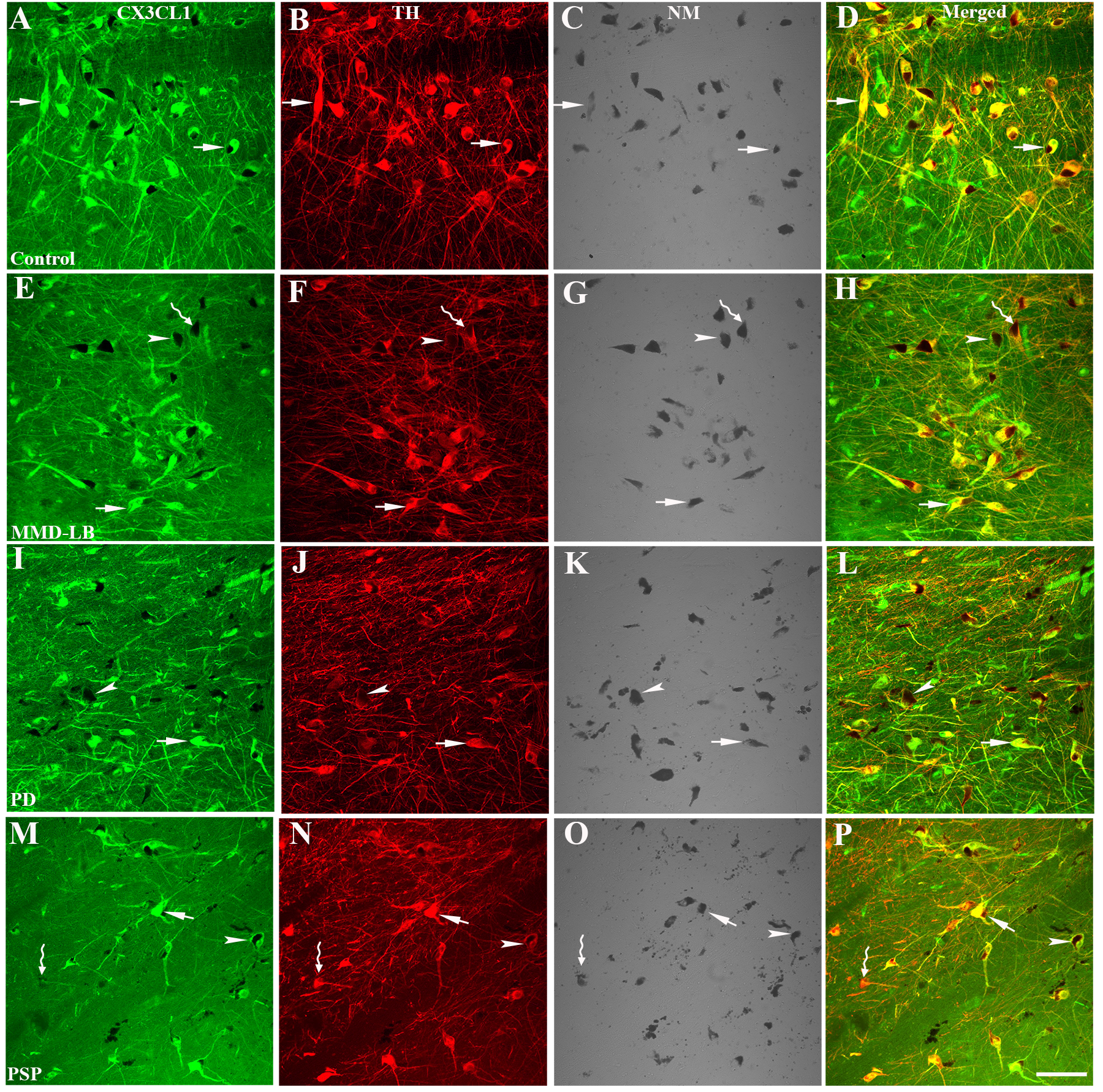

Supplement: SUPPLEMENTARY FIGURE 3 — Co-localization analyses of CX3CL1 and TH in the substantia nigra. Confocal microscopic images of the substantia nigra from age-matched control (control; A–D), MMD-LB (E–H), PD (I–L), and PSP (M–P) brains illustrated CX3CL1 (green; A,E,I,M), TH (red; B,F,J,N), neuromelanin (NM, black; C,G,K,O), and merged CX3CL1 and TH (D,H,L,P) immunofluorescent staining. Arrows denote NM-laden neurons with intensive CX3CL1 and TH double labeling (yellow; D,H,L,P). Curved arrows indicate NM-laden neurons with TH staining and absent CX3CL1. Arrowheads denote NM-laden neurons absent CX3CL1 and TH labeling. Scale bar in P = 100 μm (applies to all). [file Image_3.tif]

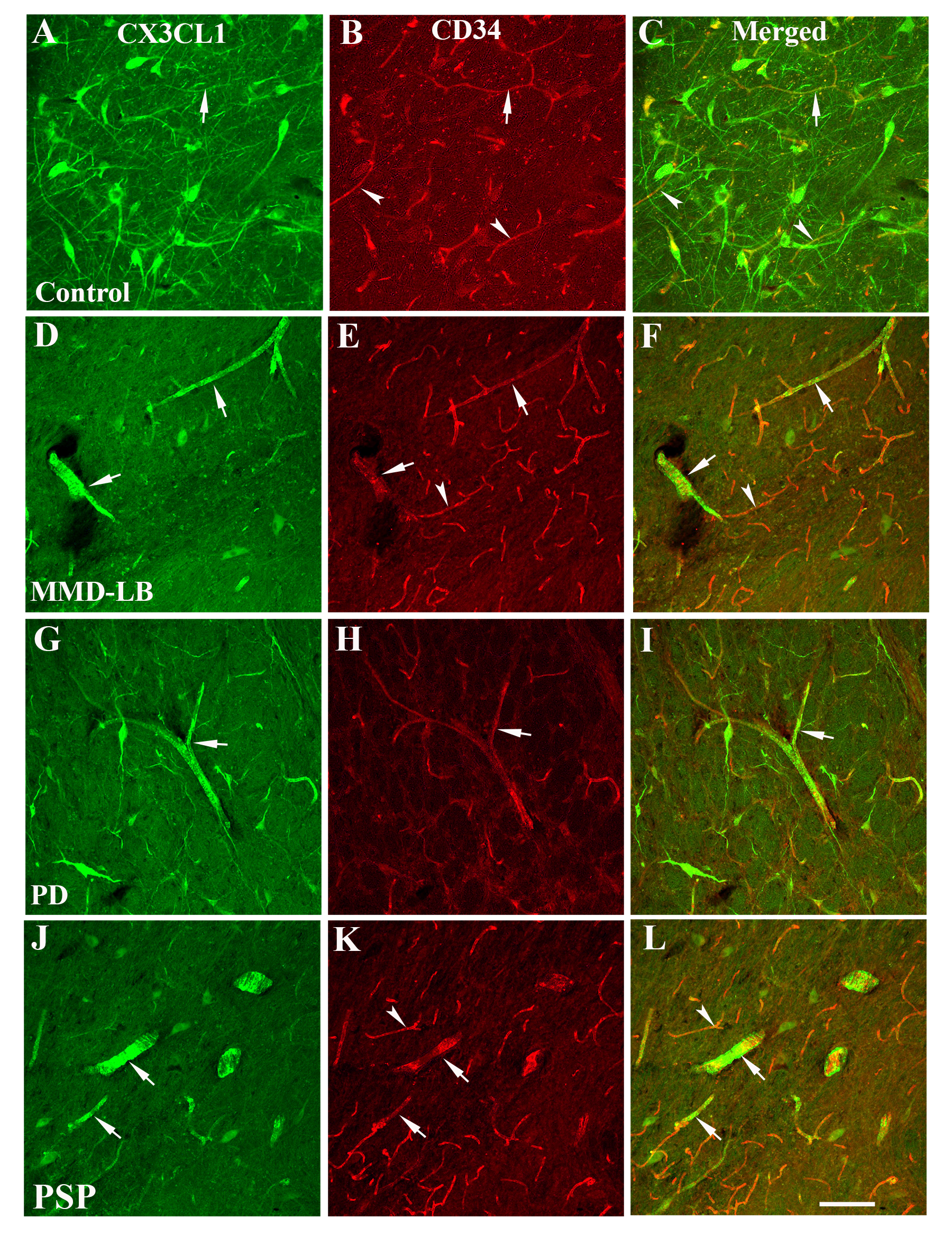

Supplement: SUPPLEMENTARY FIGURE 4 — Co-localization analyses of CX3CL1 and CD34 in the substantia nigra. Confocal microscopic images of the substantia nigra from age-matched control (control; A–C), MMD-LB (D–F), PD (G–I), and PSP (J–L) brains illustrated CX3CL1 (green; A,D,G,J), CD34 (red; B,E,H,K), and merged CX3CL1 and CD34 (C,F,I,L) immunofluorescent staining. Arrows denote blood vessels exhibiting intensive CX3CL1 and CD34 labeling (yellow; C,F,I,L). Arrowheads denote blood vessels displaying only CD34 labeling (red; C,F,I,L). Scale bar in L = 100 μm (applies to all). [file Image_10.tif]

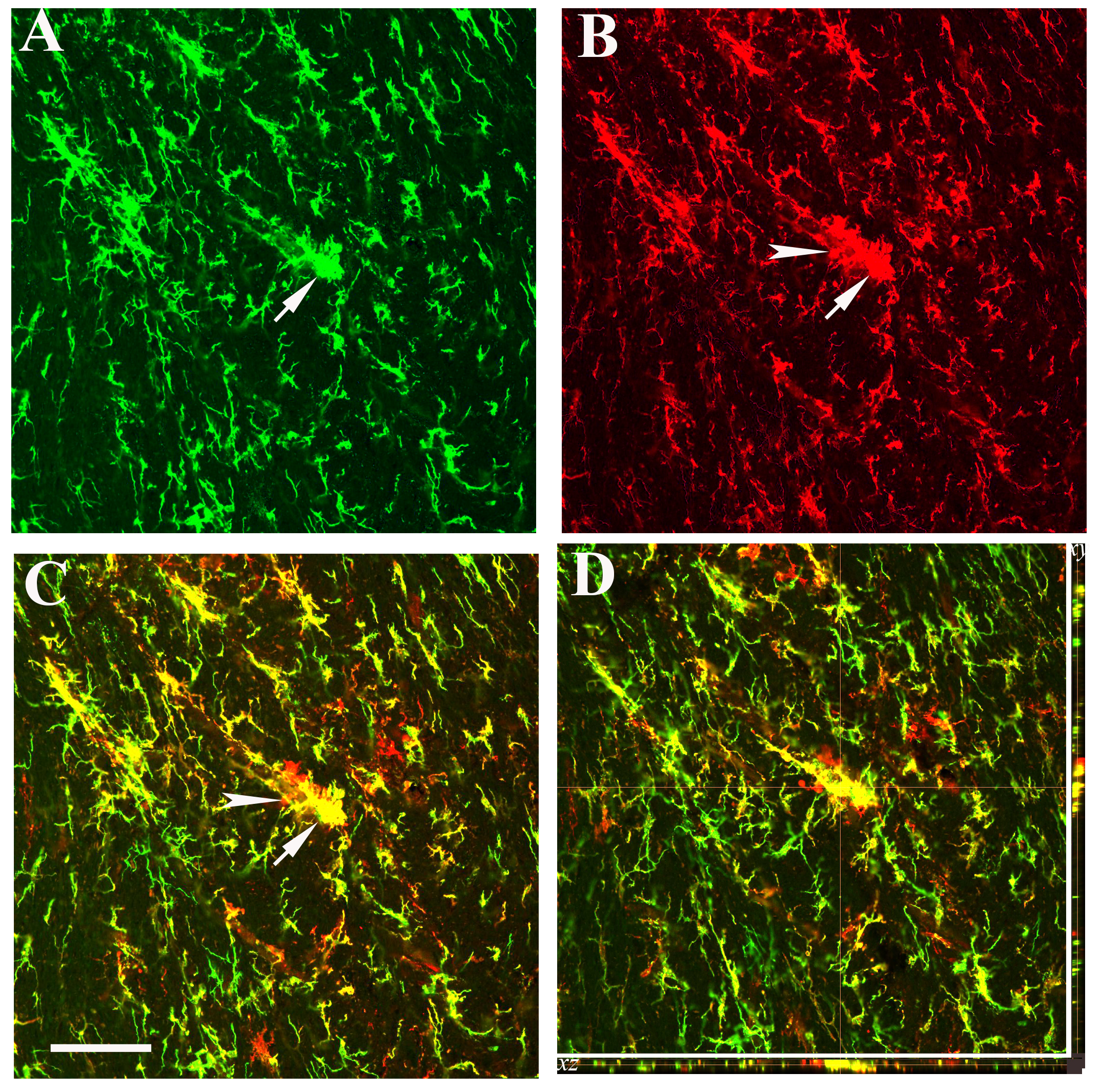

Supplement: SUPPLEMENTARY FIGURE 5 — Co-localization analyses of TMEM119 and HLA-DR in the substantia nigra. Confocal microscopic images of the substantia nigra from PD brain illustrated TMEM119 (green; A), HLA-DR (red; B), and merged TMEM119 and HLA-DR (C) immunofluorescent staining. Arrows denote enlarged microglial cell with both extensive TMEM119 and HLA-DR staining (yellow; C). Arrowheads denote HLA-DR single labeling (red; B,C). A cross-section of confocal images further illustrated the colocalization of labeled TMEM119 and HLA-DR (D): the large panel represents a cross-section of the cell layer. The horizontal and vertical lines through them denote the planes of the adjoining xz and yz sections, respectively. At the bottom and right, the xz and yz cross-sections were obtained from the combined serial optical sections of these cell layers using Nikon A1 software. The cross-section analyses revealed that TMEM119 was colocalized with HLA-DR. Scale bar in C = 100 μm (applies to all). [file Image_11.tif]

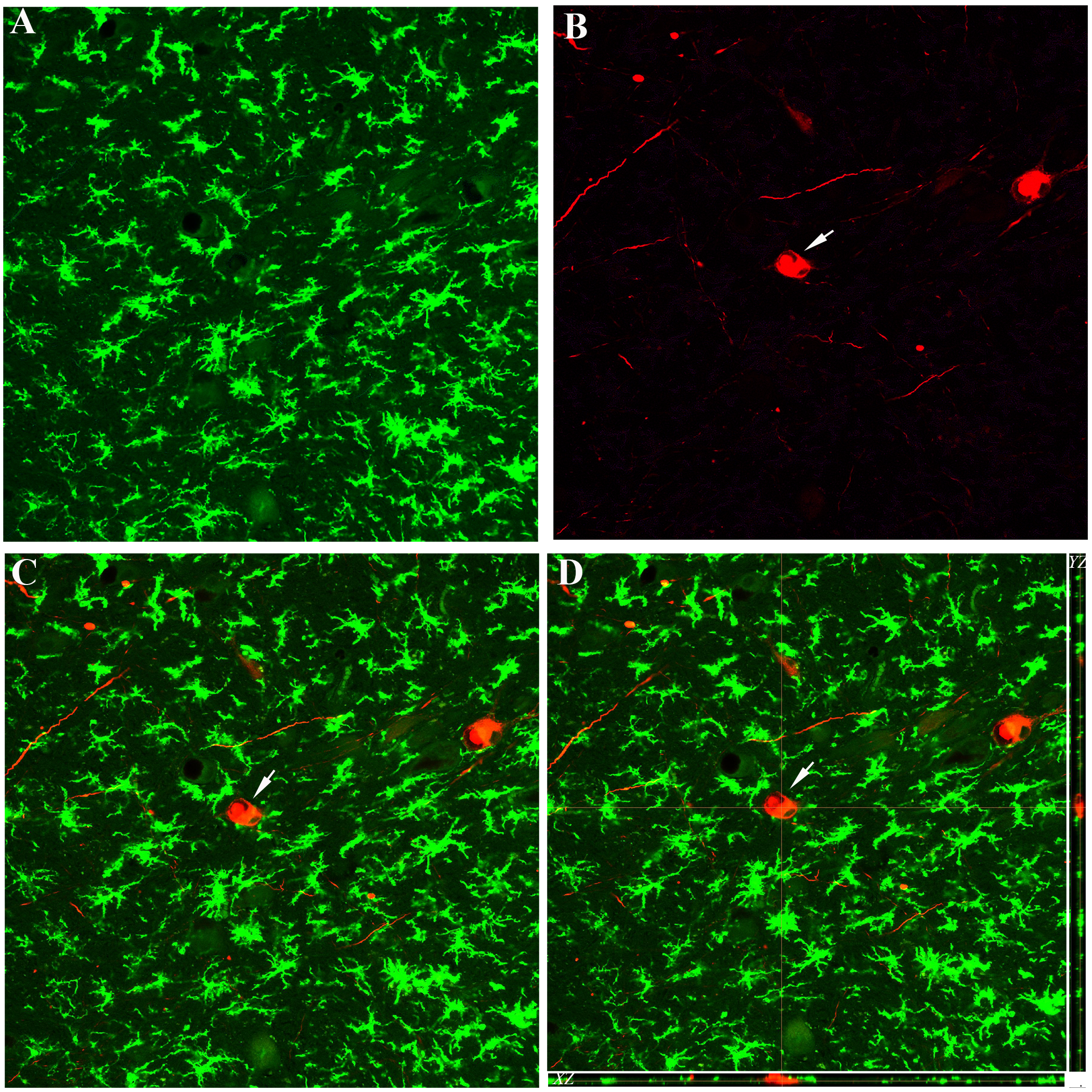

Supplement: SUPPLEMENTARY FIGURE 6 — Co-localization analyses of TMEM119 and α-synuclein (p-S129) in the substantia nigra. Confocal microscopic images of the substantia nigra from MMD-LB brain illustrated TMEM119 (green; A), p-S129 (arrow, red; B), and merged TMEM119 and p-S129 (C) immunofluorescent staining. Arrows denote p-S129 inclusion. A cross-section of confocal images further illustrated the colocalization of labeled TMEM119 and p-S129 (D): the large panel represents a cross-section of the cell layer. The horizontal and vertical lines through them denote the planes of the adjoining xz and yz sections, respectively. At the bottom and right, the xz and yz cross-sections were obtained from the combined serial optical sections of these cell layers using Nikon A1 software. The cross-section analyses revealed that TMEM119 was not colocalized with p-S129. Scale bar in C = 100 μm (applies to all). [file Image_12.tif]

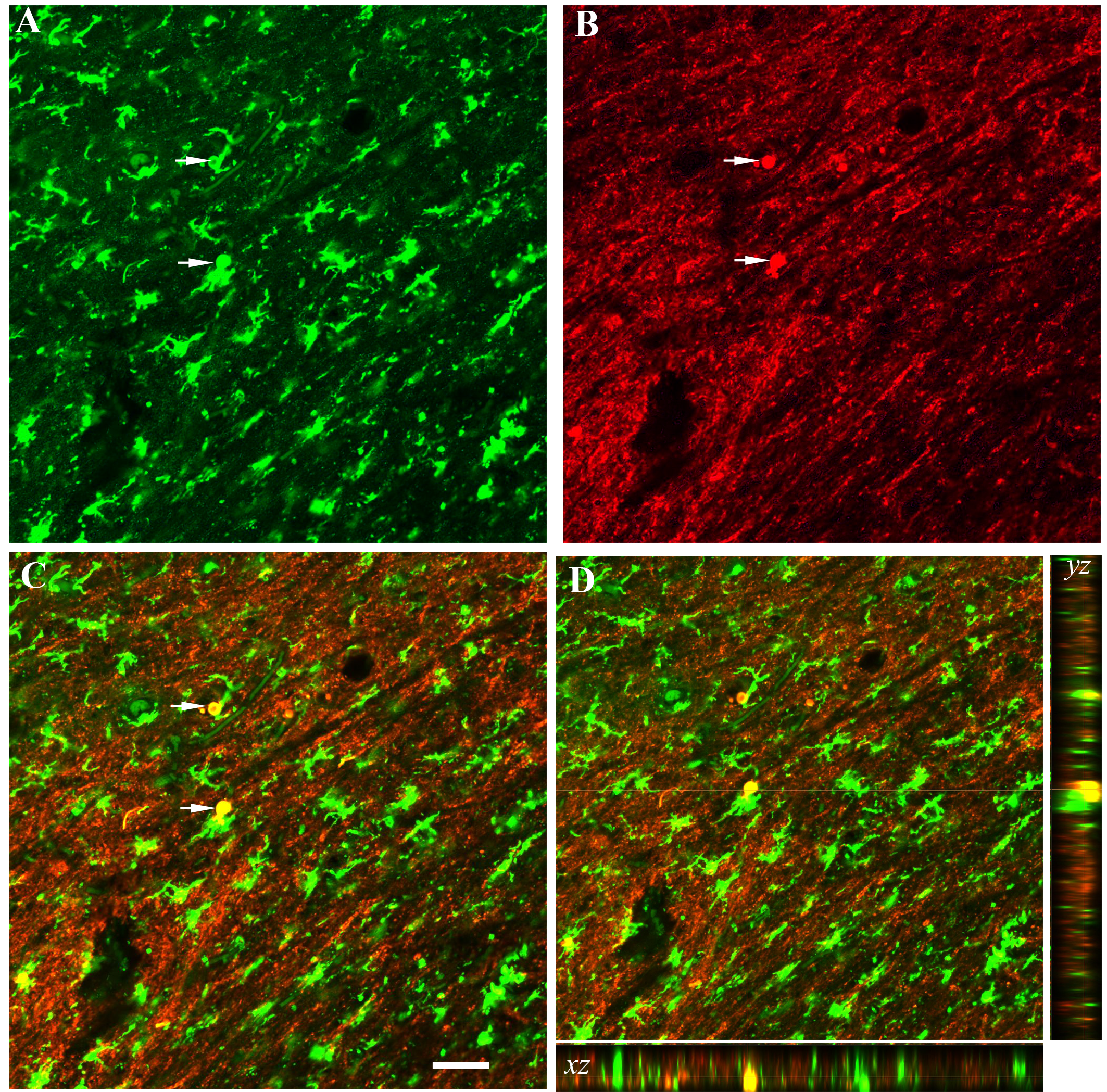

Supplement: SUPPLEMENTARY FIGURE 7 — Co-localization analyses of TMEM119 and α-synuclein (Syn05) in the substantia nigra. Confocal microscopic images of the substantia nigra from PD brain illustrated TMEM119 (green; A), Syn05 (red; B), and merged TMEM119 and Syn05 (C) immunofluorescent staining. Arrows denote the colocalization of TMEM119 and Syn05 (yellow; C). A cross-section of confocal images further illustrated the colocalization of labeled TMEM119 and Syn05 (D): the large panel represents a cross-section of the cell layer. The horizontal and vertical lines through them denote the planes of the adjoining xz and yz sections, respectively. At the bottom and right, the xz and yz cross-sections were obtained from the combined serial optical sections of these cell layers using Nikon Confocal software. The cross-section analyses revealed that a few TMEM119 were colocalized with Syn05 (D; yellow). Scale bar in C = 100 μm (applies to all). [file Image_13.tif]

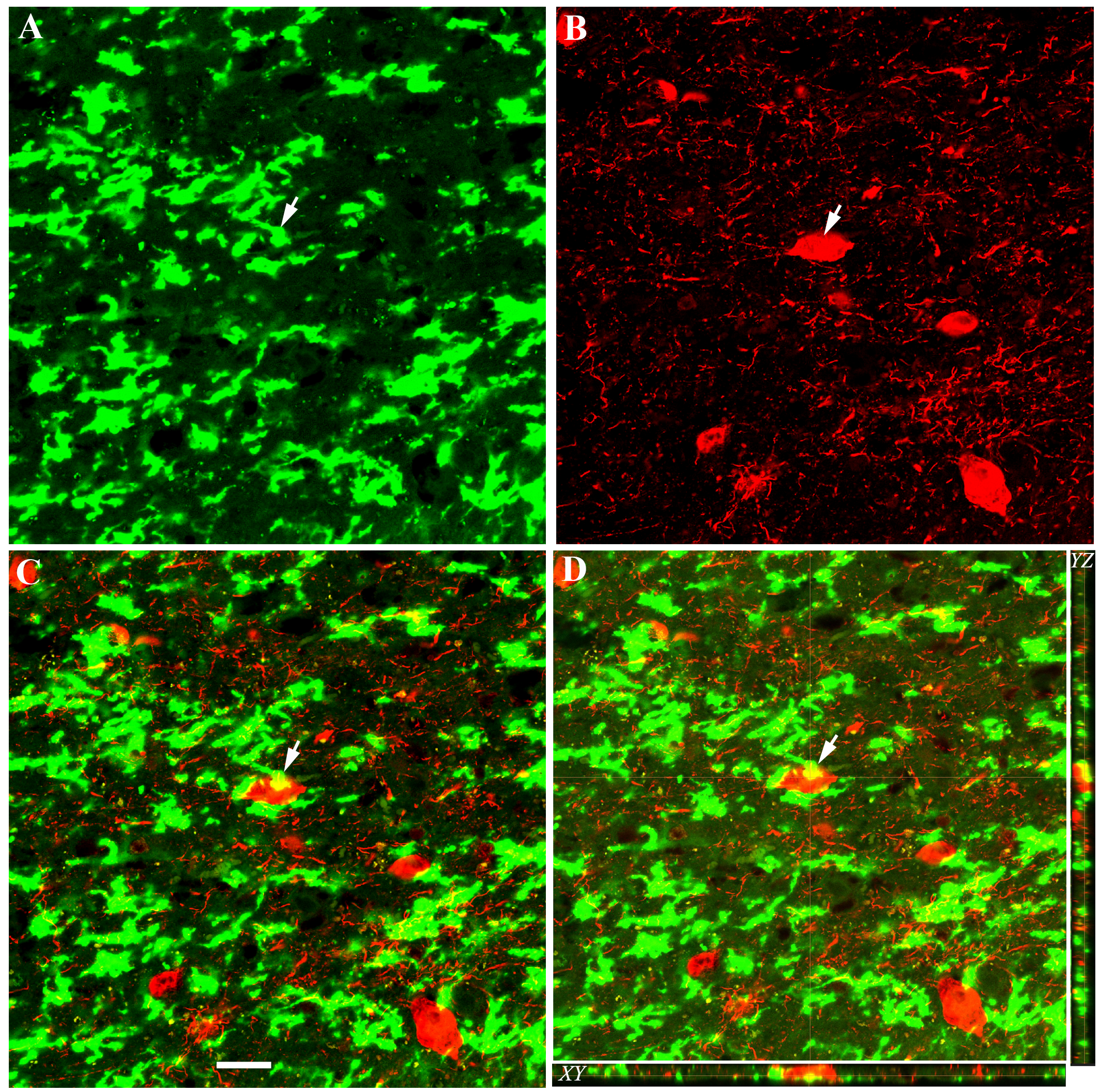

Supplement: SUPPLEMENTARY FIGURE 8 — Co-localization analyses of TMEM119 and tau (AT8) in the substantia nigra. Confocal microscopic images of the substantia nigra from PSP brain illustrated TMEM119 (green; A), AT8 (red; B), and merged TMEM119 and AT8 (C) immunofluorescent staining. Arrows denote colocalization of TMEM119 and AT8. A cross-section of the confocal image further illustrated the colocalization of labeled TMEM119 and AT8 (D): the large panel represents a cross-section of the cell layer. The horizontal and vertical lines through them denote the planes of the adjoining xz and yz sections, respectively. At the bottom and right, the xz and yz cross-sections were obtained from the combined serial optical sections of these cell layers using Nikon Confocal software. The cross-section analyses revealed that TMEM119-ir microglia attached to tau inclusions (D; yellow). Scale bar in C = 100 μm (applies to all). [file Image_14.tif]

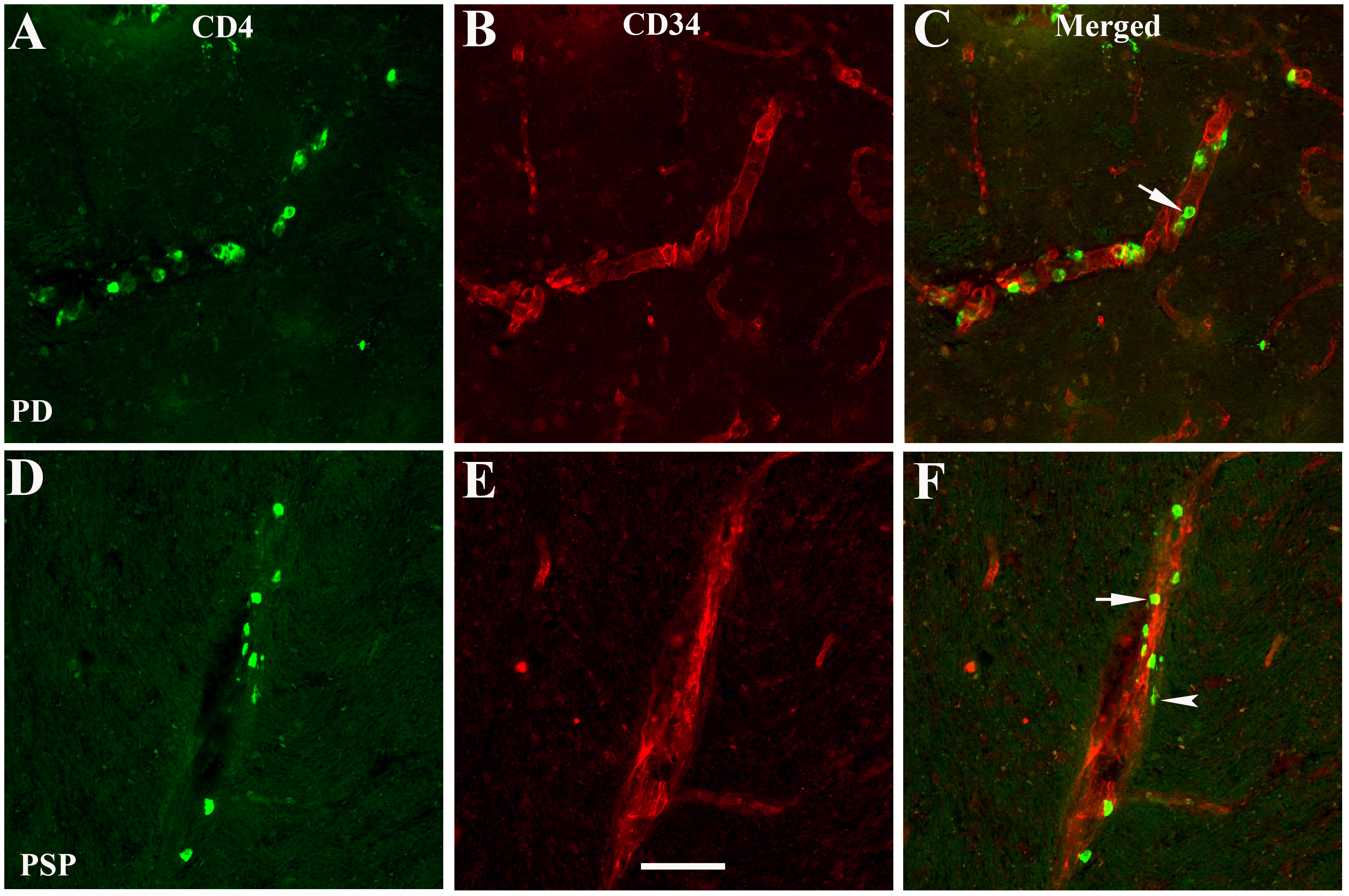

Supplement: SUPPLEMENTARY FIGURE 9 — Co-localization analyses of CD4 and CD34 in the substantia nigra. Confocal microscopic images of the substantia nigra from PD (A–C) and PSP (D–F) brains illustrated CD4 (green; A,D), CD34 (red; B,E), and merged CD4 and CD34 (C) immunofluorescent staining. Arrows denote CD4+ cells within blood vessels, and the arrowhead denotes CD4+ cells within perivascular regions. Scale bar in E = 100 μm (applies to all). [file Image_15.tif]

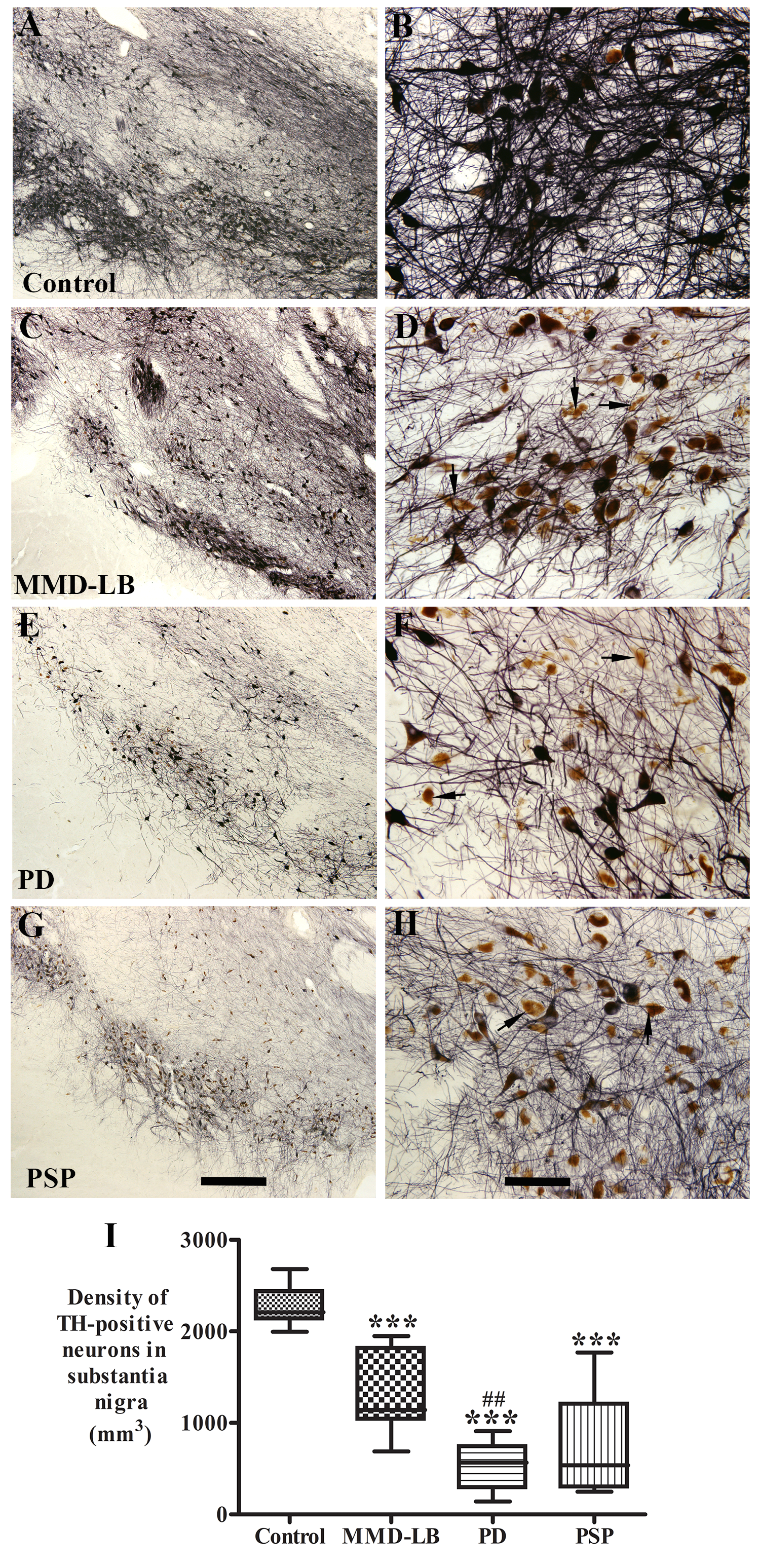

Supplement: SUPPLEMENTARY FIGURE 10 — Qualitative and quantitative evaluation of nigral TH expressions. Photomicrographs of the substantia nigra from age-matched control (control; A,B), MMD-LB (C,D), PD (E,F), and PSP (G,H) brains illustrate TH immunoreactivity. Intense TH-ir soma with an extensive local plexus of TH-ir processes was distributed throughout the substantia nigra (A,B) in the control group. Nigral TH immunoreactivity, including somata and processes, was severely reduced in disease groups (C–H) compared with control (A,B), and some remaining melanized neurons exhibited no detectable TH immunoreactivity (arrows, brown; D,F,H). Scale bar = 500 μm in G (applies to A,C,E) and 100 μm in H (applies to B,D,F). (I) Stereological analyses revealed that the density of TH-ir neurons was significantly reduced in the MMD-LB (n = 8), PD (n = 13), and PSP (n = 9) groups relative to the control (n = 8) groups. One-way ANOVA followed by Tukey’s multiple comparison test; ***p < 0.001 compared with control; ##p < 0.01 compared with MMD-LB. Stereological data from five equispaced midbrain sections in each subject. The distance between sections was approximately 0.72 mm. An optical fractionator unbiased sampling design was used to estimate TH-positive cell numbers and Cavalieri’s principle was used to estimate the volume within the substantia nigra. The densities of TH-positive neurons were calculated using the estimated TH-positive cell number from the optical fractionator/substantia nigra volume from the Cavalieri estimator (neuronal number/mm3). [file Image_16.tif]
