# Supplementary material for: Decreased neuronal and increased endothelial fractalkine expression are associated with neuroinflammation in Parkinson’s disease and related disorders
Source: Front Cell Neurosci. 2025 Aug 6;19:1557645. doi: 10.3389/fncel.2025.1557645 (PMC12364955; doi:10.3389/fncel.2025.1557645)
Supplement: Supplementary file 11 [file Table_1.docx]

**Table S1. Results of linear regression analysis**

Parameter Case Number *r p*

Age vs CX3CL1-ir neuronal number 38 0.39 0.015*

Age vs neuronal CX3CL1-ir intensity 30 0.19 0.29

Age vs TMEM119-ir microglial number 38 0.12 0.93

Age vs TMEM119-ir microglial area 38 0.16 0.25 UPDRS III vs CX3CL1-ir neuronal number 18 0.031 0.90

UPDRS III vs CX3CL1-ir neuronal intensity 18 0.085 0.73

UPDRS III vs TMEM119-ir microglial number 21 0.306 0.17

UPDRS III vs TMEM119-ir microglial area 21 0.485 0.026*

H&Y vs CX3CL1-ir neuronal number 18 0.081 0.74

H&Y vs CX3CL1-ir neuronal intensity 18 0.149 0.56

H&Y vs TMEM119-ir microglial number 21 0.049 0.83

H&Y vs TMEM119-ir microglial area 22 0.138 0.53

CD4+ T cell number vs TMEM119-ir microglial number 34 0.261 0.13

UPDRS III: Unified Parkinson’s disease rating scale part III, H&Y: Hoehn and Yahr.
